# Identifying crosstalk genetic biomarkers linking a neurodegenerative disease, Parkinson’s disease, and periodontitis using integrated bioinformatics analyses

**DOI:** 10.3389/fnagi.2022.1032401

**Published:** 2022-12-05

**Authors:** Shaonan Hu, Simin Li, Wanchen Ning, Xiuhong Huang, Xiangqiong Liu, Yupei Deng, Debora Franceschi, Anthony Chukwunonso Ogbuehi, Bernd Lethaus, Vuk Savkovic, Hanluo Li, Sebastian Gaus, Rüdiger Zimmerer, Dirk Ziebolz, Gerhard Schmalz, Shaohong Huang

**Affiliations:** ^1^Stomatological Hospital, Southern Medical University, Guangzhou, China; ^2^Laboratory of Molecular Cell Biology, Beijing Tibetan Hospital, China Tibetology Research Center, Beijing, China; ^3^Department of Experimental and Clinical Medicine, University of Florence, Florence, Italy; ^4^Faculty of Physics, University of Münster, Münster, Germany; ^5^Department of Cranio Maxillofacial Surgery, University Clinic Leipzig, Leipzig, Germany; ^6^Department of Cariology, Endodontology and Periodontology, University of Leipzig, Leipzig, Germany

**Keywords:** neurodegenerative disease, Parkinson’s disease, periodontitis, crosstalk genes, bioinformatics

## Abstract

**Objective:**

To identify the genetic linkage mechanisms underlying Parkinson’s disease (PD) and periodontitis, and explore the role of immunology in the crosstalk between both these diseases.

**Methods:**

The gene expression omnibus (GEO) datasets associated with whole blood tissue of PD patients and gingival tissue of periodontitis patients were obtained. Then, differential expression analysis was performed to identify the differentially expressed genes (DEGs) deregulated in both diseases, which were defined as crosstalk genes. Inflammatory response-related genes (IRRGs) were downloaded from the MSigDB database and used for dividing case samples of both diseases into different clusters using k-means cluster analysis. Feature selection was performed using the LASSO model. Thus, the hub crosstalk genes were identified. Next, the crosstalk IRRGs were selected and Pearson correlation coefficient analysis was applied to investigate the correlation between hub crosstalk genes and hub IRRGs. Additionally, immune infiltration analysis was performed to examine the enrichment of immune cells in both diseases. The correlation between hub crosstalk genes and highly enriched immune cells was also investigated.

**Results:**

Overall, 37 crosstalk genes were found to be overlapping between the PD-associated DEGs and periodontitis-associated DEGs. Using clustering analysis, the most optimal clustering effects were obtained for periodontitis and PD when k = 2 and k = 3, respectively. Using the LASSO feature selection, five hub crosstalk genes, namely, FMNL1, MANSC1, PLAUR, RNASE6, and TCIRG1, were identified. In periodontitis, MANSC1 was negatively correlated and the other four hub crosstalk genes (FMNL1, PLAUR, RNASE6, and TCIRG1) were positively correlated with five hub IRRGs, namely, AQP9, C5AR1, CD14, CSF3R, and PLAUR. In PD, all five hub crosstalk genes were positively correlated with all five hub IRRGs. Additionally, RNASE6 was highly correlated with myeloid-derived suppressor cells (MDSCs) in periodontitis, and MANSC1 was highly correlated with plasmacytoid dendritic cells in PD.

**Conclusion:**

Five genes (i.e., FMNL1, MANSC1, PLAUR, RNASE6, and TCIRG1) were identified as crosstalk biomarkers linking PD and periodontitis. The significant correlation between these crosstalk genes and immune cells strongly suggests the involvement of immunology in linking both diseases.

## Introduction

Parkinson’s disease (PD) is a chronic, debilitating, neurodegenerative disorder with both motor and nonmotor symptoms that gradually worsen over time (Blaszczyk, 2016). Systemic inflammatory response plays a critical role in the progression of PD by activating microglial cells and initiating the cascade of neurodegeneration (Kaur et al., 2016). Periodontitis is a highly prevalent, multifactorial, chronic inflammatory disease of the periodontium, which causes destruction of the supportive tissues of the teeth and, eventually, tooth loss (Kaur et al., 2018). Several previous epidemiological studies have indicated a possible link between periodontal disease and PD. Some studies have reported that PD patients have a higher prevalence and risk of developing periodontal disease compared to a person without PD (Schwarz et al., 2006; Hanaoka and Kashihara, 2009). The cognitive dysfunction of PD patients prevented them from effectively performing routine daily oral hygiene activities (Al-Omari et al., 2014). Additionally, one of the side effects of multiple medications was altered salivary flow rates, thereby affecting self-cleaning oral mechanisms (Auffret et al., 2021). In other studies, periodontitis was found to be associated with an increased risk of developing PD (Chen et al., 2017). The ulcerations in the periodontal pocket lining provided easy access for periodontal pathogens (e.g., *Aggregatibacter actinomycetemcomitans* (Aa), *Porphyromonas gingivalis* (Pg), *Tannerella forsythia* (Tf), *Treponema denticola* (Td), and *Fusobacterium nucleatum* (Fn)) and inflammatory mediators (e.g., pro-inflammatory cytokines) to infiltrate the systemic peripheral blood circulation (Scannapieco, 2004). Lipopolysaccharide (LPS), an endotoxin and component of the Gram-negative bacterial cell wall, caused the breakdown of the blood brain barrier (BBB) and activated the microglial cells, which led to the necrosis and apoptosis of dopaminergic neurons in the substantia nigra (SN) of the midbrain (Bohatschek et al., 2001; Olsen et al., 2020).

Apart from inflammatory response, systemic immune activation may be another link connecting periodontitis and PD. It is suggested that periodontitis triggers the systemic immune response, following which the activated immune cells from the peripheral blood overcome the BBB and promote central nervous system (CNS) inflammation in PD patients (Zhang et al., 2021). Further, certain genetic susceptibility factors have been demonstrated to increase the risk of both periodontitis and PD (Tettamanti et al., 2017; Karimi-Moghadam et al., 2018). A previous study conducted by João Botelho and colleagues analyzed the Genome-Wide Association Studies (GWAS) data and identified the protein variants strongly associated with PD and periodontitis onset (Botelho et al., 2020). However, it may be worthy to note that a potential limitation of this research is that its study design was not from an inflammatory or immunological perspective. Thus, to fill this gap, our current study aimed to explore the missing genetic links between PD and periodontitis, particularly focusing on inflammatory response-associated genes and immune cells.

Many previous studies used computational biology approaches to explore the shared genetic linkages between two pathogenesis-related diseases (Li et al., 2018; Chen et al., 2021, 2022; He et al., 2021; Alves et al., 2022; Pan et al., 2022; Yan et al., 2022; Liu et al., 2022a,b). In order to investigate the shared genetic links between PD and periodontitis, integrated bioinformatics analyses were also utilized. First, differentially expressed genes (DEGs) deregulated in both diseases were obtained, and crosstalk genes were identified. The hub crosstalk genes were selected by performing k-means cluster analysis based on inflammatory response-related genes (IRRGs). Then, the correlation between hub crosstalk genes and IRRGs as well as the correlation between hub crosstalk genes and immune cells were investigated.

## Materials and methods

### Study design of the current research

**Figure 1:**
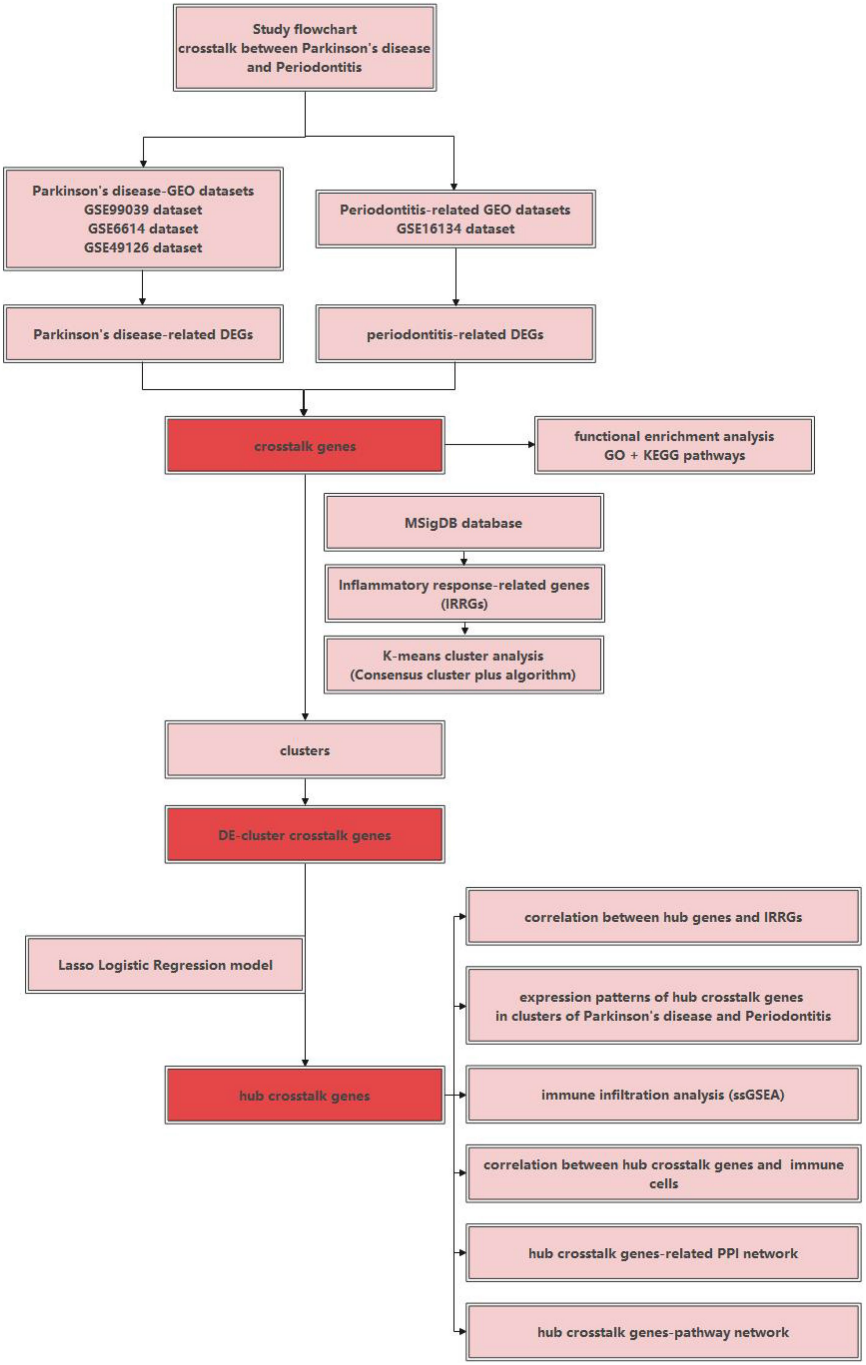
The study flowchart of the current research.

Figure 1 used a schematic diagram to show the study flowchart of the current research. Firstly, differential expression analysis was performed to identify the DEGs belonging to PD and periodontitis respectively, and thus crosstalk genes were identified. Secondly, IRRGs were obtained and used for performing the K-means cluster analysis, and then differentially expressed (DE)- cluster crosstalk genes were identified. Thirdly, hub crosstalk genes were identified by building the Lasso logistic regression model. Lastly, hub crosstalk genes as the investigation focus were researched from many aspects, for example, correlation with IRRGs; expression patterns of hub crosstalk genes in clusters of both diseases; correlation with immune cells; related PPI network; related pathway network.

### Datasets

The datasets related to periodontitis and PD were, respectively, downloaded from the gene expression omnibus (GEO)^1^ database (Edgar et al., 2002). The inclusion criteria for the periodontitis-related GEO datasets are as below: (1) The study design should be set as: established periodontitis samples as the experimental group and healthy gingival samples as the control group. (2) Periodontitis was defined in accordance with the case definition presented in the 2017 World Workshop: ① interdental CAL detectable at ≥2 nonadjacent teeth or ② buccal or oral with CAL ≥3 mm with pocketing >3 mm detectable at ≥2 teeth (Tonetti et al., 2018). (3) The sample size of the disease samples should be more than 20. The inclusion criteria for the PD-related GEO datasets are as below: (1) The study design should aim to compare the transcriptomic profiling of the blood samples (e.g., whole blood, peripheral blood mononuclear cells) between PD patients and healthy control subjects. (2) The sample size of the disease samples should be more than 20. Following such inclusion criteria, the GSE16134 dataset (Papapanou et al., 2009; Kebschull et al., 2014) was selected to be a periodontitis-related GEO dataset, and three datasets [GSE6613 (Scherzer et al., 2007, 2008), GSE49126 (Mutez et al., 2014), and GSE99039 (Shamir et al., 2017)] were selected to be PD-related datasets. Table 1 shows detailed information about these four included datasets.

**Table 1:** Datasets of periodontitis and Parkinson’s disease.

	Periodontitis		Parkinson’s	
Series	GSE16134	GSE99039	GSE6613	GSE49126
Platform	GPL570	GPL570	GPL96	GPL4133
**Tissue**	**Gingival**		**Whole blood**	
Case	241	205	50	30
Control	69	233	22	20
Total sample	310	438	72	50

By downloading the human gene set “HALLMARK_INFLAMMATORY_RESPONSE”^2^ from the MSigDB database^3^ (Lopez et al., 2018), 200 IRRGs were obtained. In addition, the gene signatures of 28 tumor-infiltrating lymphocytes were downloaded from the TISIDB database^4^ (Zhang et al., 2013).

### Data preprocessing

The Probe ID in the expression matrix was converted to the gene symbol based on the respective platform information for the downloaded data. If one Probe ID corresponds to multiple gene symbols, the gene symbols were de-duplicated based on the average of the sample expression values. When the number of samples with a gene of 0 in the expression matrix exceeds half of the total number of samples, the gene was removed from the expression matrix. In addition, Log2 was calculated for the dataset with large sample expression values, in order to achieve data standardization.

For PD, three datasets related to PD (i.e., GSE6613, GSE49126, GSE99039) were combined based on a common Gene Symbol. These three datasets were designed under different experimental platforms: GSE6613—GPL96 [HG-U133A] Affymetrix Human Genome U133A Array; GSE49126—GPL4133 Agilent-014850 Whole Human Genome Microarray 4x44K G4112F (Feature Number version); GSE99039—GPL570 [HG-U133_Plus_2] Affymetrix Human Genome U133 Plus 2.0 Array. After merging, the ComBat funtion in the “sva” package (version 4.1.3) (Leek et al., 2019) was used to eliminate the batch effect of the merged data. The PCA analysis was carried out, respectively, on the combined expression matrix and the expression matrix after ComBat correction, in order to examine the effects after ComBat correction.

### Differential expression analysis

The “limma” package (version 3.15) (Ritchie et al., 2015) was used to analyze the differential expression between Case-sample and Normal-sample and identify the DEGs. DEGs were identified according to different thresholds of P.adjust and log2FC (log2 fold change) for periodontitis dataset and PD dataset, respectively. The P.adjust<0.05 and | log2FC | > 0.5 were selected as the threshold for selecting DEGs in periodontitis dataset. The P.adjust<0.05 and | log2FC | > 0.15 was selected as the threshold when selecting DEGs in PD datasets.

### Cross-talk gene between periodontitis disease and PD

To explore potential relationships between Periodontitis disease and PD, DEGs deregulated in periodontitis and DEGs deregulated in PD were obtained respectively, and their intersection genes were labelled as cross-talk genes. These intersection genes could be considered as the bridge genes connecting the relationship between periodontitis and PD. The “pheatmap” package (version 1.0.12) (Kolde and Kolde, 2018) was used to analyze the expression level of these cross-talk genes. The “ClusterProfiler” package (version 3.15) (Yu et al., 2012; Wu et al., 2021) in R was utilized to analyze the cross-talk gene for Gene Ontology (GO) term-biological processes (BPs) and KEGG pathways to observe the biological functions affected by these cross-talk genes.

### Consensus cluster plus analysis of periodontitis and Parkinson’s based on the IRRG dataset

The differentially expressed IRRGs were obtained from periodontitis and PD, respectively. Then the expression values of the differentially expressed IRRGs in case samples were obtained for periodontitis and PD, respectively. The Consensus Cluster Plus algorithm (Wilkerson and Hayes, 2010) was utilized to carry out k-means cluster analysis based on the expression matrix of case samples.

Before using the Consensus Cluster Plus algorithm, it is important to determine the number of clusters. To determine the optimal number of samples, the interval silhouette width and elbow method were used to analyze the IRRGs expression matrix of periodontitis and PD, respectively. The fviz_nbclust method of “factoextra” package (version 1.0.7) (Kassambara and Mundt, 2017) was used for the analysis. The average silhouette width automatically calculated the number of the optimum clusters. The elbow method was used to observe the slope change of the vertical within sum of square (WSS). When the WSS decreases very slowly, increasing the number of clusters can no longer increase the clustering effect, and the existence of this “elbow point” is the optimal number of clusters. The optimal number of clusters was determined by considering the results of the two methods together. The “ConsensusClusterPlus” algorithm was used, and optimum cluster value was defined as the value of the parameter maxK. The case samples in periodontitis and PD were divided into different clusters based on IRRGs. Afterward, the expression levels of IRRG in different clusters were analyzed.

### Identification of hub cross-talk genes based on cluster

The expression values of cross-talk gene in the case sample of the two diseases were obtained, and then “limma” package (version 3.15) (Ritchie et al., 2015) was used to analyze the cross-talk gene differentially expressed in different clusters according to the respective cluster categories. The cross-talk genes of p.adjust <0.01 were labeled as DE-cluster cross-talk genes. The least absolute shrinkage and selection operator (LASSO) Logistic Regression approach was used to further filter the DE-cluster cross-talk gene.

The expression values of the DE-cluster cross-talk gene in the respective disease’s case samples were obtained, and then LASSO model was used for feature screening according to the respective cluster. According to the lambda.min value in the analysis results, the feature genes of both diseases were obtained. Finally, the intersection of the feature genes of the two diseases were obtained, and these intersection genes were regarded as the final hub cross-talk genes. These hub cross-talk genes were not only differentially expressed in periodontitis and PD, but also had a potential relationship with IRRG.

### The correlation between hub cross-talk genes and IRRGs

To observe the relationship between hub cross-talk genes and IRRGs, the overlapping between IRRGs and cross-talk genes were obtained and considered as the IRR cross-talk genes. The expression values of hub cross-talk genes and IRR cross-talk genes in the case sample of two diseases were obtained. The Pearson correlation coefficient analysis was used to analyze the relationship among hub cross-talk genes, as well as the correlation between hub cross-talk genes and hub IRRGs.

### Hub cross-talk gene expression analysis

The expression matrix of hub cross-talk genes in periodontitis and PD were obtained, and the Wilcoxon test was performed to examine the significance of hub cross-talk genes. Meanwhile, the expression matrix of the hub cross-talk genes under different clusters were obtained. Periodontitis data set has two clusters, and Wilcoxon test was used to examine the significance of hub cross-talk genes. PD-related data set had three clusters, and Kruskal test was used to examine the significance of hub cross-talk genes. ROC analysis was carried out to investigate the predictive effects of hub cross-talk genes, based on the expression matrices of these genes in case-samples and control-samples.

### Immune infiltrates

After downloading the immune cell-related data from TISDB database, we performed ssGSEA quantitative analysis was performed to calculate the abundance of immune cells in both diseases. The “GSVA” package (version 3.15) (Hänzelmann et al., 2013) was used in this analysis. The enriched and abundant immune cells were obtained through hierarchical clustering. The correlation between the abundant immune cells and other immune cells was analyzed by using Pearson correlation coefficient analysis. The Wilcoxon test was performed to analyze the differences of the abundance levels of these immune cells between case samples and control samples.

### Correlation between immune cells and hub cross-talk genes

The fractions of abundant immune cells in the case samples and the expression of hub cross-talk gene in the case samples were obtained. The correlation between immune cells and the hub cross-talk genes were analyzed by using the Pearson correlation coefficient analysis.

### Hub cross-talk gene PPI network and pathway network

The protein–protein interaction (PPI) relationship pair between the Hub cross-talk gene and other genes were extracted from the human protein reference database (HPRD)^5^ (Peri et al., 2004) and the biological general repository for interaction datasets (BIOGRID)^6^ (Oughtred et al., 2021). These interaction pairs were regarded as the hub cross-talk genes-target interaction pairs. The relevant genes under all pathways were procured from the Kyoto Encyclopedia of Genes and Genomes (KEGG) database^7^ (Kanehisa and Goto, 2000). The hub cross-talk gene-pathway relationship pair was firstly obtained. Afterward, the pathway-target relationship pair was obtained according to the pathways in the hub cross-talk gene-pathway interaction pairs. Therefore the hub cross-talk gene-pathway-target interaction pairs were identified, based on which the complex network of Hub cross-talk gene-Target and Hub cross-talk gene-Pathway-Target were constructed by using Cytoscape software (version 3.8) (Shannon et al., 2003).

## Results

### Data preprocessing

**Figure 2:**
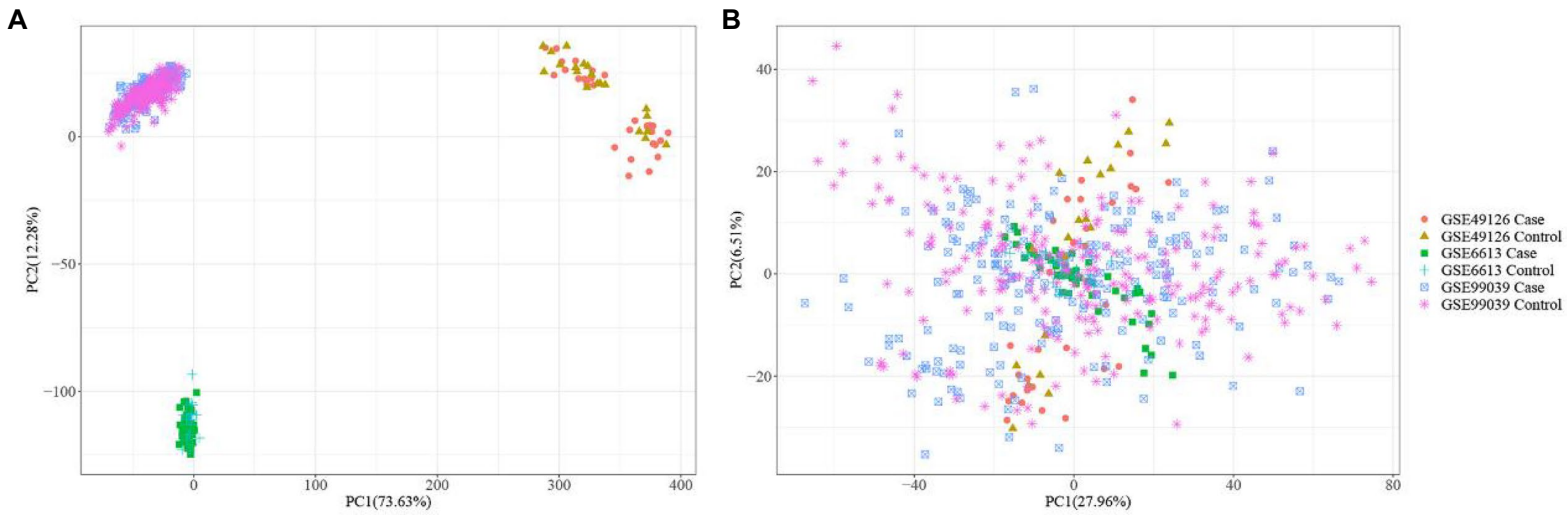
PCA analysis plots before **(A)** and after batch correction **(B)** of Parkinson’s disease related dataset.

The three datasets related to PD (i.e., GSE6613, GSE49126, GSE99039) were merged, and then the PCA analysis was performed (Figure 2A). Figure 2A shows that the 3 datasets differed significantly. Figure 2B shows that the batch effect generated by merging datasets was significantly eliminated. By comparing Figures 2A,B, it was found that the differences between the three datasets were significantly reduced.

### Differentially expressed genes

Differential expression analysis was conducted for the periodontitis and PD-related datasets. For periodontitis-related dataset (GSE16134), the genes of P.adjust < 0.05 and |log2FC| > 0.5 were selected as DEGs, where log2FC >0.5 was the upregulated DEGs and log2FC < −0.5 was the downregulated DEGs. For PD-related datasets (GSE6613, GSE49126, GSE99039), the genes of P.adjust < 0.05 and | log2FC| > 0.15 were selected as DEGs, where log2FC > 0.15 were upregulated DEGs and log2FC < −0.15 were down-regulated DEGs. Table 2 shows the number of upregulated and downregulated DEGs deregulated in periodontitis and PD.

**Table 2:** DEG statistical results.

Datasets	Periodontitis		Parkinson’s	
	GSE16134	GSE6613	GSE49126	GSE99039
P.adjust	P.adj < 0.05		P.adj < 0.05	
|Log2(FC)|	|Log2(FC)| > 0.5		|Log2(FC)| > 0.15	
DEG up	795		199	
DEG down	546		12	
Total DEG	1,341		211	

**Figure 3:**
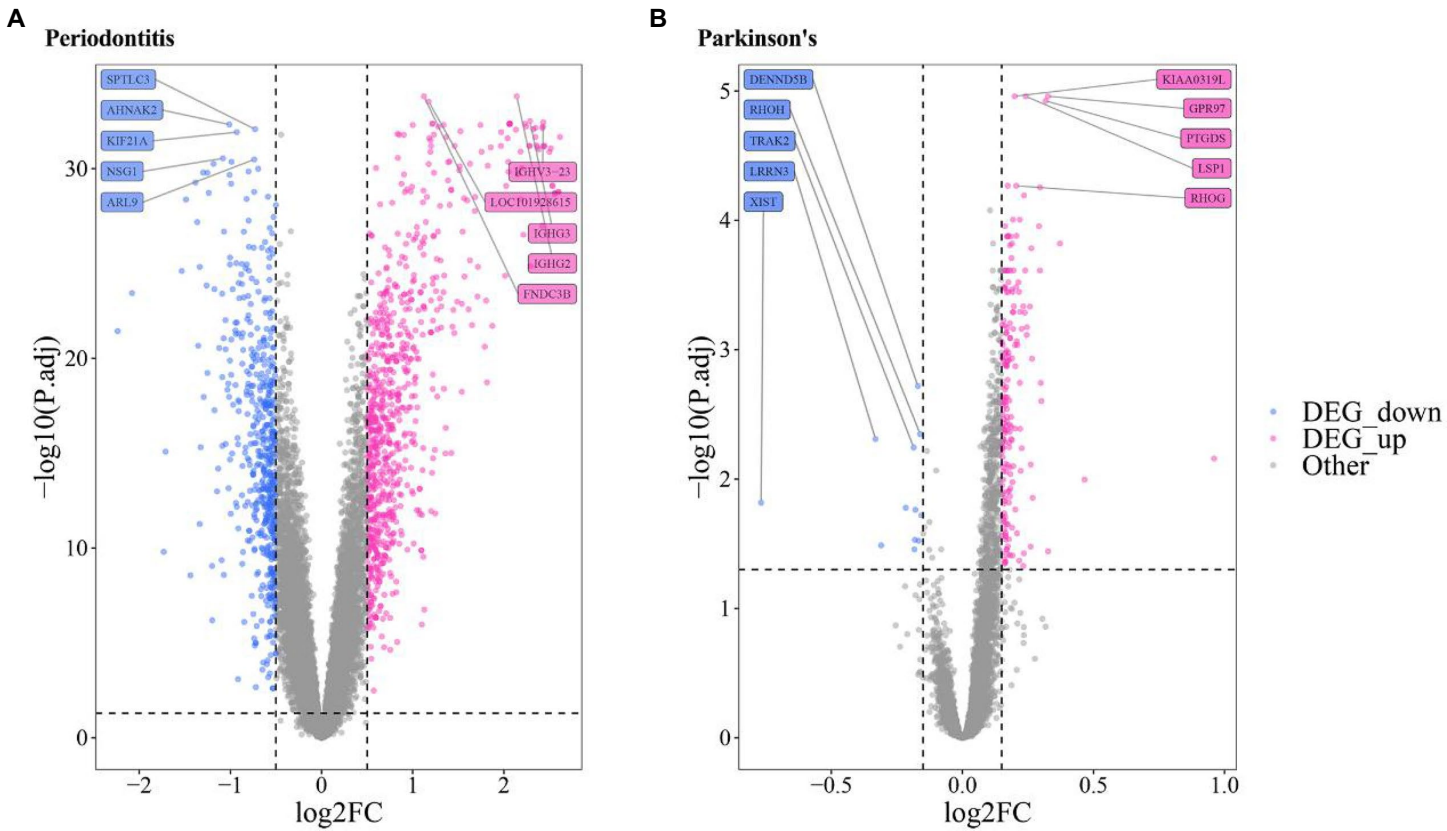
Volcanic map of the differentially expressed gene of **(A)** Periodontitis disease **(A)** and **(B)** Parkinson’s disease.

A volcano map was utilized to show the distribution of DEGs in Periodontitis- (Figure 3A) and PD-related data (Figure 3B). The top5 up-regulated and down-regulated DEGs with the most significant P.adjust values were labeled.

### Cross-talk gene

**Figure 4:**
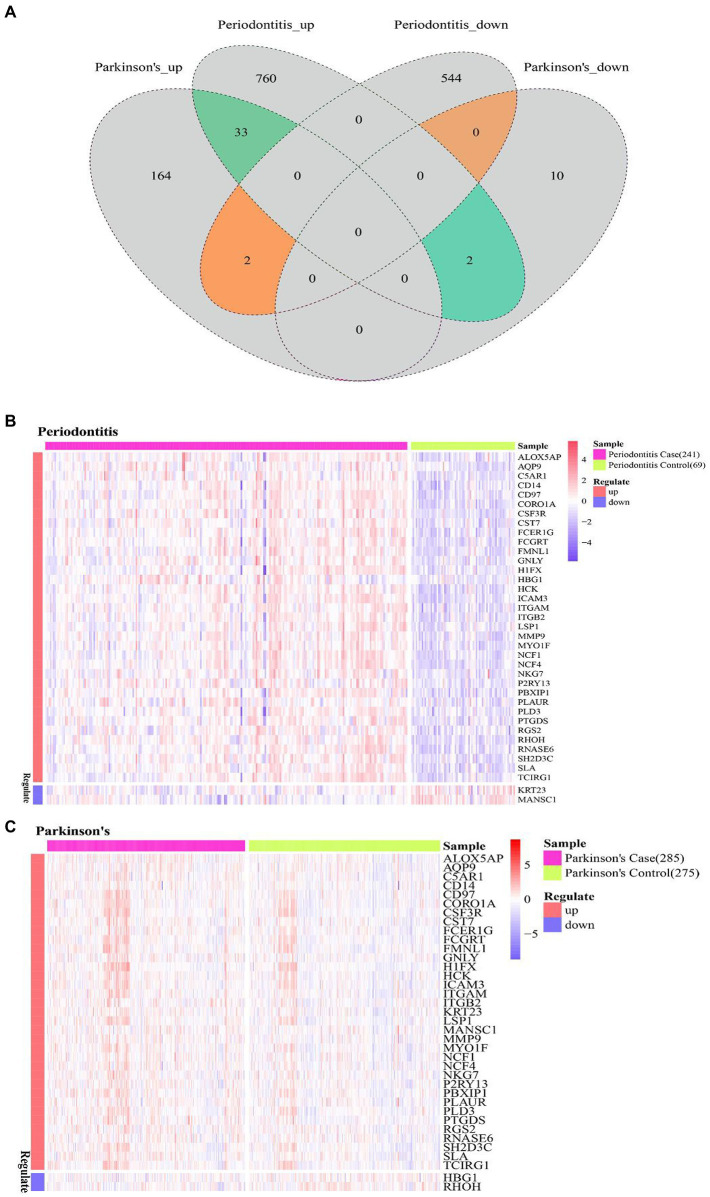
Cross-talk gene expression level. **(A)** Venn diagram of periodontitis disease and Parkinson’s disease differentially expressed genes; **(B,C)** cross-talk gene at the level of expression of Periodontitis disease and Parkinson’s disease.

The common DEGs of Periodontitis disease and PD were extracted, and thus 37 Cross-talk genes were obtained (Figure 4A). Then the expression values of 37 cross-talk genes in periodontitis and PD were extracted, base on which heatmaps were plotted for periodontitis (Figure 4B) and PD (Figure 4C).

**Figure 5:**
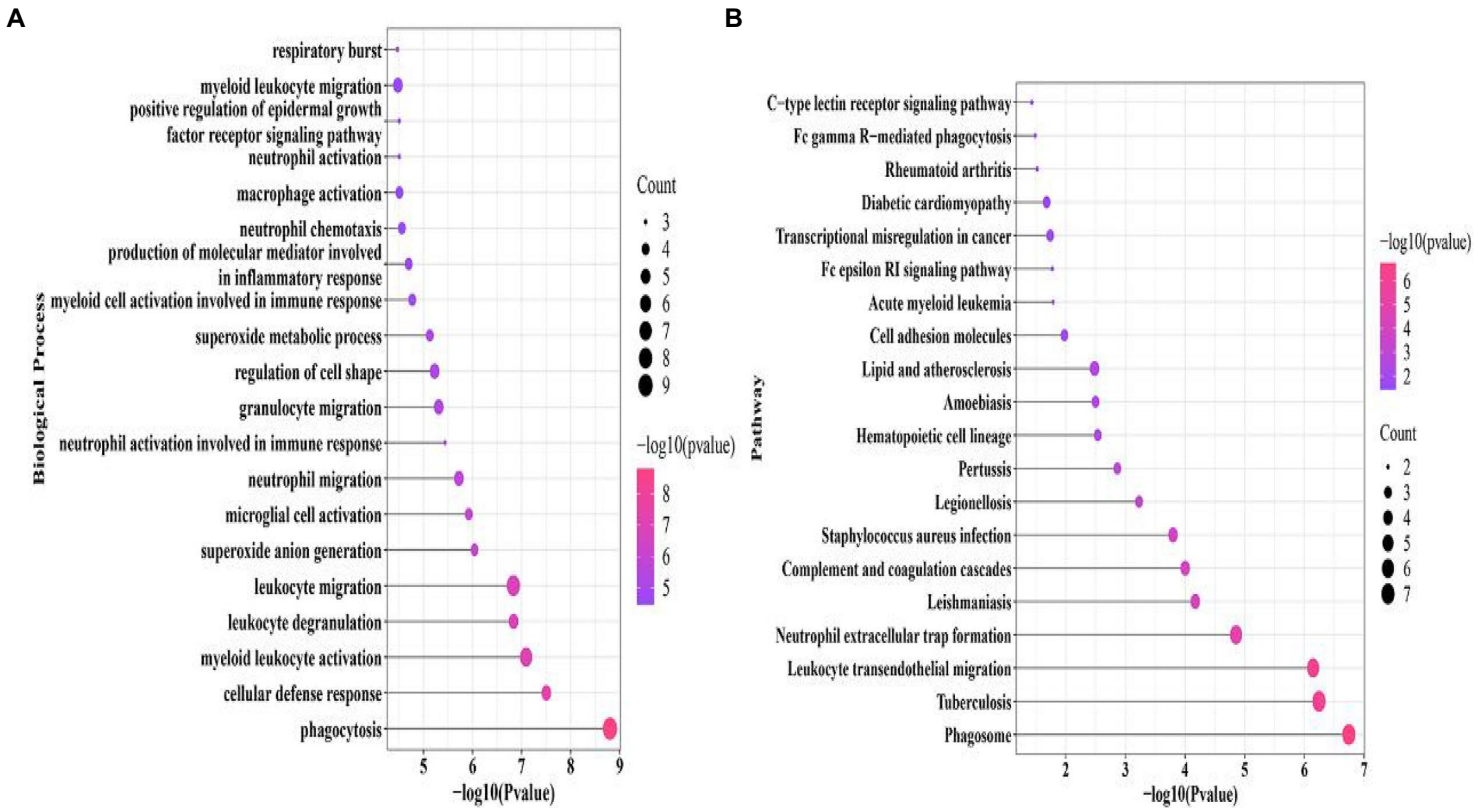
Cross-talk gene significant enrichment of biological processes **(A)** and pathway **(B)**.

The “ClusterProfiler” package in R was used to identify the BPs and KEGG pathways enriched by 37 cross-talk genes. The *p* < 0.05 was established as statistical significance and the top 20 terms were visualized (Figures 5A,B; Tables 3, 4).

**Table 3:** The top 20 biological processes enriched by the crosstalk genes.

ID	Description	GeneRatio	BgRatio	*p*-value	P.adjust	*q*-value	geneID	Count
GO:0006909	Phagocytosis	9/32	308/18723	1.58E-09	1.46E-06	9.79E-07	929/11151/2207/3055/3385/3684/3689/4689/399	9
GO:0006968	Cellular defense response	5/32	54/18723	3.13E-08	1.45E-05	9.71E-06	728/10578/4046/653361/10312	5
GO:0002274	Myeloid leukocyte activation	7/32	223/18723	8.09E-08	2.49E-05	1.67E-05	728/8530/2207/3684/3689/5730/399	7
GO:0043299	Leukocyte degranulation	5/32	73/18723	1.45E-07	2.71E-05	1.82E-05	11,151/3055/3684/3689/5730	5
GO:0050900	Leukocyte migration	8/32	369/18723	1.47E-07	2.71E-05	1.82E-05	728/11151/1441/2207/3055/3689/4318/399	8
GO:0042554	Superoxide anion generation	4/32	44/18723	9.09E-07	0.000140002	9.39E-05	3684/3689/653361/4689	4
GO:0001774	Microglial cell activation	4/32	47/18723	1.19E-06	0.000157107	0.000105418	728/8530/3684/3689	4
GO:1990266	Neutrophil migration	5/32	122/18723	1.89E-06	0.000218547	0.000146644	728/1441/2207/3689/399	5
GO:0002283	Neutrophil activation involved in immune response	3/32	18/18723	3.64E-06	0.000373357	0.000250521	2207/3684/3689	3
GO:0097530	Granulocyte migration	5/32	148/18723	4.89E-06	0.000451796	0.000303153	728/1441/2207/3689/399	5
GO:0008360	Regulation of cell shape	5/32	154/18723	5.94E-06	0.000498738	0.000334651	11,151/752/3055/3689/399	5
GO:0006801	Superoxide metabolic process	4/32	74/18723	7.43E-06	0.000572401	0.000384078	3684/3689/653361/4689	4
GO:0002275	Myeloid cell activation involved in immune response	4/32	91/18723	1.69E-05	0.001202578	0.000806925	2207/3684/3689/5730	4
GO:0002532	Production of molecular mediator involved in inflammatory response	4/32	95/18723	2.01E-05	0.001323779	0.00088825	241/653361/57326/23646	4
GO:0030593	Neutrophil chemotaxis	4/32	103/18723	2.76E-05	0.001475695	0.000990185	728/1441/2207/3689	4
GO:0042116	Macrophage activation	4/32	106/18723	3.09E-05	0.001475695	0.000990185	728/8530/3684/3689	4
GO:0042119	Neutrophil activation	3/32	36/18723	3.12E-05	0.001475695	0.000990185	2207/3684/3689	3
GO:0045742	Positive regulation of epidermal growth factor receptor signaling pathway	3/32	36/18723	3.12E-05	0.001475695	0.000990185	4318/653361/5329	3
GO:0097529	Myeloid leukocyte migration	5/32	220/18723	3.33E-05	0.001475695	0.000990185	728/1441/2207/3689/399	5
GO:0045730	Respiratory burst	3/32	37/18723	3.39E-05	0.001475695	0.000990185	3055/653361/4689	3

**Table 4:** The top 20 signaling pathways enriched by the crosstalk genes.

ID	Description	GeneRatio	BgRatio	*p-*value	p.adjust	*q*-value	geneID	Count
hsa04145	Phagosome	7/24	152/8160	1.80E-07	1.31E-05	1.00E-05	929/11151/3684/3689/653361/4689/10312	7
hsa05152	Tuberculosis	7/24	180/8160	5.70E-07	1.74E-05	1.33E-05	929/11151/2207/3684/3689/4046/10312	7
hsa04670	Leukocyte transendothelial migration	6/24	114/8160	7.14E-07	1.74E-05	1.33E-05	3684/3689/4318/653361/4689/399	6
hsa04613	Neutrophil extracellular trap formation	6/24	190/8160	1.40E-05	0.000255023	0.000194899	366/728/3684/3689/653361/4689	6
hsa05140	Leishmaniasis	4/24	77/8160	6.75E-05	0.000985329	0.000753027	3684/3689/653361/4689	4
hsa04610	Complement and coagulation cascades	4/24	85/8160	9.94E-05	0.001209374	0.000924251	728/3684/3689/5329	4
hsa05150	Staphylococcus aureus infection	4/24	96/8160	0.000159591	0.00166431	0.001271931	728/3684/3689/25984	4
hsa05134	Legionellosis	3/24	57/8160	0.000589439	0.005378627	0.004110558	929/3684/3689	3
hsa05133	Pertussis	3/24	76/8160	0.00136523	0.01107353	0.008462828	929/3684/3689	3
hsa04640	Hematopoietic cell lineage	3/24	99/8160	0.002913961	0.020016567	0.015297448	929/1441/3684	3
hsa05146	Amoebiasis	3/24	102/8160	0.003171487	0.020016567	0.015297448	929/3684/3689	3
hsa05417	Lipid and atherosclerosis	4/24	215/8160	0.003290395	0.020016567	0.015297448	929/4318/653361/4689	4
hsa04514	Cell adhesion molecules	3/24	157/8160	0.010513557	0.059037667	0.045118909	3385/3684/3689	3
hsa05221	Acute myeloid leukemia	2/24	67/8160	0.016316475	0.081667353	0.062413406	929/3684	2
hsa04664	Fc epsilon RI signaling pathway	2/24	68/8160	0.016780963	0.081667353	0.062413406	241/2207	2
hsa05202	Transcriptional misregulation in cancer	3/24	193/8160	0.018292556	0.083459789	0.063783256	929/3684/4318	3
hsa05415	Diabetic cardiomyopathy	3/24	203/8160	0.020897732	0.089737318	0.068580791	4318/653361/4689	3
hsa05323	Rheumatoid arthritis	2/24	93/8160	0.030142526	0.12224469	0.093424204	3689/10312	2
hsa04666	Fc gamma R-mediated phagocytosis	2/24	97/8160	0.032573664	0.125151447	0.095645662	3055/653361	2
hsa04625	C-type lectin receptor signaling pathway	2/24	104/8160	0.037008335	0.135080423	0.103233777	2207/4046	2

The cross-talk genes were mainly involved in several BPs, for example, cellular defense response, leukocyte degranulation, neutrophil migration, and neutrophil activation involved in immune response (Figure 5A; Table 3). In addition, cross-talk genes regulate several KEGG pathways, for instance, phagotsome, leukocyte transendothelial migration, neutrophil extracellular trap formation, staphylococcus aureus infection, cell adhesion molecules, and Fc epsilon RI Signaling pathways (Figure 5B; Table 4).

**Table 5:** The correlation among hub crosstalk genes in periodontitis and Parkinson’s disease, respectively.

Hub gene	Hub gene	Cor.	*p*-value	Sig.
**Periodontitis**				
FMNL1	TCIRG1	0.776680579	8.72E-64	***
TCIRG1	FMNL1	0.776680579	8.72E-64	***
FMNL1	RNASE6	0.630304404	9.84E-36	***
RNASE6	FMNL1	0.630304404	9.84E-36	***
RNASE6	TCIRG1	0.623645546	8.28E-35	***
TCIRG1	RNASE6	0.623645546	8.28E-35	***
FMNL1	PLAUR	0.519504303	7.95E-23	***
PLAUR	FMNL1	0.519504303	7.95E-23	***
PLAUR	TCIRG1	0.463852082	6.06E-18	***
TCIRG1	PLAUR	0.463852082	6.06E-18	***
PLAUR	RNASE6	0.356920531	9.59E-11	***
RNASE6	PLAUR	0.356920531	9.59E-11	***
MANSC1	PLAUR	−0.501651214	3.67E-21	***
PLAUR	MANSC1	−0.501651214	3.67E-21	***
MANSC1	RNASE6	−0.508910042	7.94E-22	***
RNASE6	MANSC1	−0.508910042	7.94E-22	***
MANSC1	TCIRG1	−0.583097828	1.25E-29	***
TCIRG1	MANSC1	−0.583097828	1.25E-29	***
FMNL1	MANSC1	−0.687320353	1.13E-44	***
MANSC1	FMNL1	−0.687320353	1.13E-44	***
**Parkinson’s disease**				
FMNL1	TCIRG1	0.714413971	1.39E-88	***
TCIRG1	FMNL1	0.714413971	1.39E-88	***
MANSC1	PLAUR	0.542333646	3.87E-44	***
PLAUR	MANSC1	0.542333646	3.87E-44	***
FMNL1	PLAUR	0.375957267	3.05E-20	***
PLAUR	FMNL1	0.375957267	3.05E-20	***
PLAUR	RNASE6	0.373867758	5.10E-20	***
RNASE6	PLAUR	0.373867758	5.10E-20	***
PLAUR	TCIRG1	0.369494651	1.48E-19	***
TCIRG1	PLAUR	0.369494651	1.48E-19	***
RNASE6	TCIRG1	0.294141212	1.22E-12	***
TCIRG1	RNASE6	0.294141212	1.22E-12	***
MANSC1	RNASE6	0.268167983	1.12E-10	***
RNASE6	MANSC1	0.268167983	1.12E-10	***
FMNL1	MANSC1	0.181137877	1.61E-05	***
MANSC1	FMNL1	0.181137877	1.61E-05	***
FMNL1	RNASE6	0.151556057	0.000319114	***
RNASE6	FMNL1	0.151556057	0.000319114	***
MANSC1	TCIRG1	0.110844015	0.008657743	**
TCIRG1	MANSC1	0.110844015	0.008657743	**

**p <= 0.01, ***p <= 0.001.

**Table 6:** The correlation between hub crosstalk genes and hub IRRGs in periodontitis and Parkinson’s disease.

Hub gene	Hub IRRG	Cor.	*p*-value	Sig.
**Periodontitis**				
RNASE6	CD14	0.764628729	1.02E-60	***
PLAUR	C5AR1	0.671344512	5.74E-42	***
TCIRG1	CD14	0.664226041	8.15E-41	***
FMNL1	CSF3R	0.645560153	6.13E-38	***
FMNL1	CD14	0.643618516	1.19E-37	***
FMNL1	C5AR1	0.590656767	1.54E-30	***
RNASE6	C5AR1	0.569100882	5.29E-28	***
TCIRG1	CSF3R	0.568078374	6.90E-28	***
RNASE6	CSF3R	0.550156788	6.35E-26	***
PLAUR	CSF3R	0.542527119	4.02E-25	***
FMNL1	PLAUR	0.519504303	7.95E-23	***
PLAUR	CD14	0.509187187	7.48E-22	***
TCIRG1	C5AR1	0.479173686	3.36E-19	***
TCIRG1	PLAUR	0.463852082	6.06E-18	***
PLAUR	AQP9	0.424911367	5.07E-15	***
RNASE6	AQP9	0.4075901	7.75E-14	***
RNASE6	PLAUR	0.356920531	9.59E-11	***
FMNL1	AQP9	0.263403908	2.57E-06	***
TCIRG1	AQP9	0.179628798	0.001494316	**
MANSC1	AQP9	−0.245665691	1.21E-05	***
MANSC1	C5AR1	−0.407813463	7.49E-14	***
MANSC1	PLAUR	−0.501651214	3.67E-21	***
MANSC1	CSF3R	−0.533100941	3.68E-24	***
MANSC1	CD14	−0.620737902	2.06E-34	***
**Parkinson’s disease**				
TCIRG1	CSF3R	0.739465803	5.77E-98	***
FMNL1	CSF3R	0.730984072	1.14E-94	***
MANSC1	C5AR1	0.723131244	9.90E-92	***
PLAUR	C5AR1	0.69290086	2.67E-81	***
MANSC1	AQP9	0.631283537	1.35E-63	***
PLAUR	AQP9	0.630607165	2.01E-63	***
MANSC1	PLAUR	0.542333646	3.87E-44	***
PLAUR	CSF3R	0.505325432	1.25E-37	***
PLAUR	CD14	0.440176835	6.11E-28	***
FMNL1	C5AR1	0.422459064	1.20E-25	***
RNASE6	AQP9	0.41039563	3.66E-24	***
FMNL1	PLAUR	0.375957267	3.05E-20	***
RNASE6	PLAUR	0.373867758	5.10E-20	***
TCIRG1	C5AR1	0.372498455	7.13E-20	***
TCIRG1	PLAUR	0.369494651	1.48E-19	***
TCIRG1	CD14	0.363230267	6.59E-19	***
RNASE6	C5AR1	0.359788021	1.48E-18	***
FMNL1	CD14	0.358011298	2.24E-18	***
RNASE6	CD14	0.355084478	4.39E-18	***
MANSC1	CSF3R	0.347787555	2.29E-17	***
MANSC1	CD14	0.324463762	3.40E-15	***
RNASE6	CSF3R	0.282898522	9.14E-12	***
TCIRG1	AQP9	0.231098723	3.17E-08	***
FMNL1	AQP9	0.205778472	9.04E-07	***

**p <= 0.01, ***p <= 0.001.

### Consensus cluster plus analysis results

**Figure 6:**
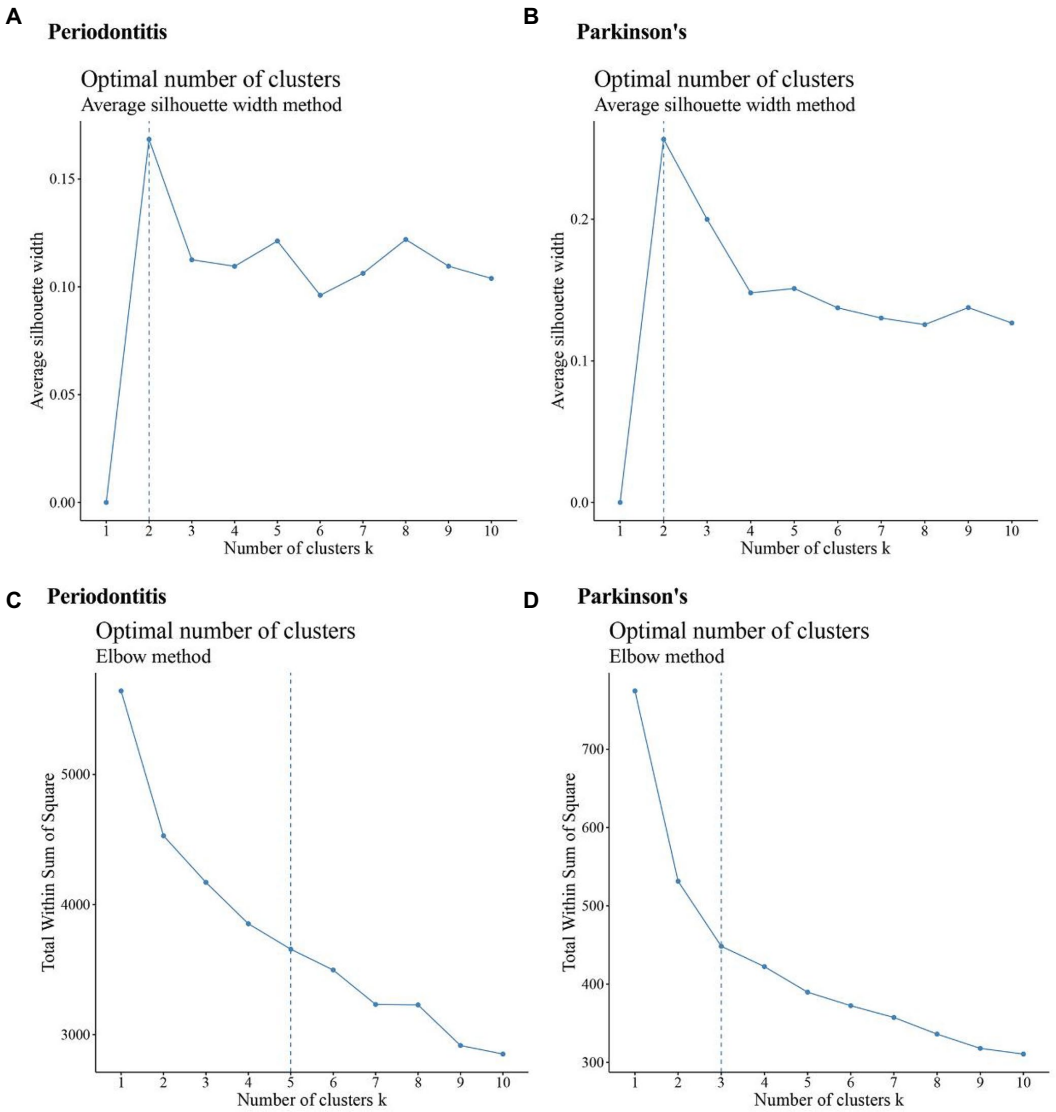
Results of a cluster analysis of periodontitis and Parkinson’s disease. **(A,B)** Results of a silhouette width analysis of the IRRGs associated with periodontitis and the average of IRRGs associated with Parkinson’s disease; **(C,D)** Results of an elbow method analysis of Periodontitis disease-related IRRGs and Parkinson’s disease-related IRRGs.

The differentially expressed IRRGs in periodontitis and PD were extracted; thereby 47 periodontitis-related IRRGs and 9 PD-related IRRGs were identified. The expression values of periodontitis-related IRRGs and PD-related IRRGs were obtained, respectively. Afterward, the IRRG-related expression profiles were analyzed by using the average silhouette width and elbow method (Figures 6A–D).

The clusters’ numbers of Periodontitis disease were determined as 2 (Figure 6A) and 5 (Figure 6C) respectively. Based on this, the maxK parameter was set as 5 when periodontitis dataset was calculated by using “Consensus Cluster Plus” function. The clusters’ numbers of PD are 2 (Figure 6B) and 3 (Figure 6D). Based on this, the maxK parameter was set as 3 when PD-related data set was calculated by using “Consensus Cluster Plus” function.

**Figure 7:**
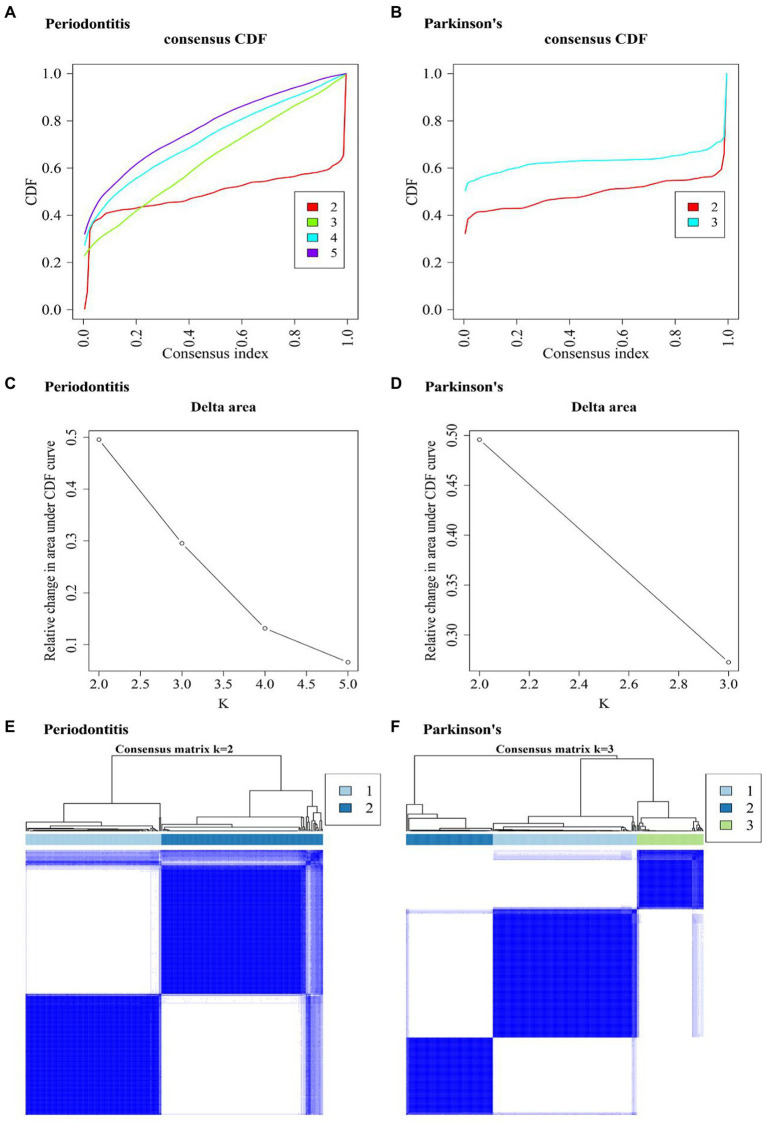
Consensus Cluster Plus analysis results. **(A,B)** Consistent cumulative distribution function (CDF) plot of periodontitis and Parkinson’s disease. This plot shows the cumulative distribution function of the score when k takes different values, which is used to determine when k is taken, CDF reaches an approximate maximum, and the cluster analysis results are the most reliable. That is, consider the k-value of the CDF descent slope is small. **(C,D)** The k and k-1 relative changes in area under the CDF curve in periodontitis and Parkinson’s disease. The total area under the CDF curve at k = 2 (the area below the line in **(A,B)** rather than the relative change in area). To select the final k-value, we should consider that the descending slope of the midline of **(A,B)** is as small as possible, and also consider that the relative change in the area under k and k-1 in **(C,D)** compared to the CDF curve is as small as possible. **(E,F)** Consistent cluster diagram of periodontitis and Parkinson’s disease.

By using “Consensus Cluster Plus” function, two diseases’ clustering results were obtained (Figures 7A–D). Figures 7A,C show that the periodontitis-related data achieved the best clustering effects when k = 2. Figures 7B,D show that the PD-related data achieved the best clustering effects when k = 3. Figures 7E,F shows the sample correlation results when k = 2 being selected for periodontitis data (Figure 7E) and when k = 3 being selected for PD data (Figure 7F).

### Hub cross-talk genes screening

**Figure 8:**
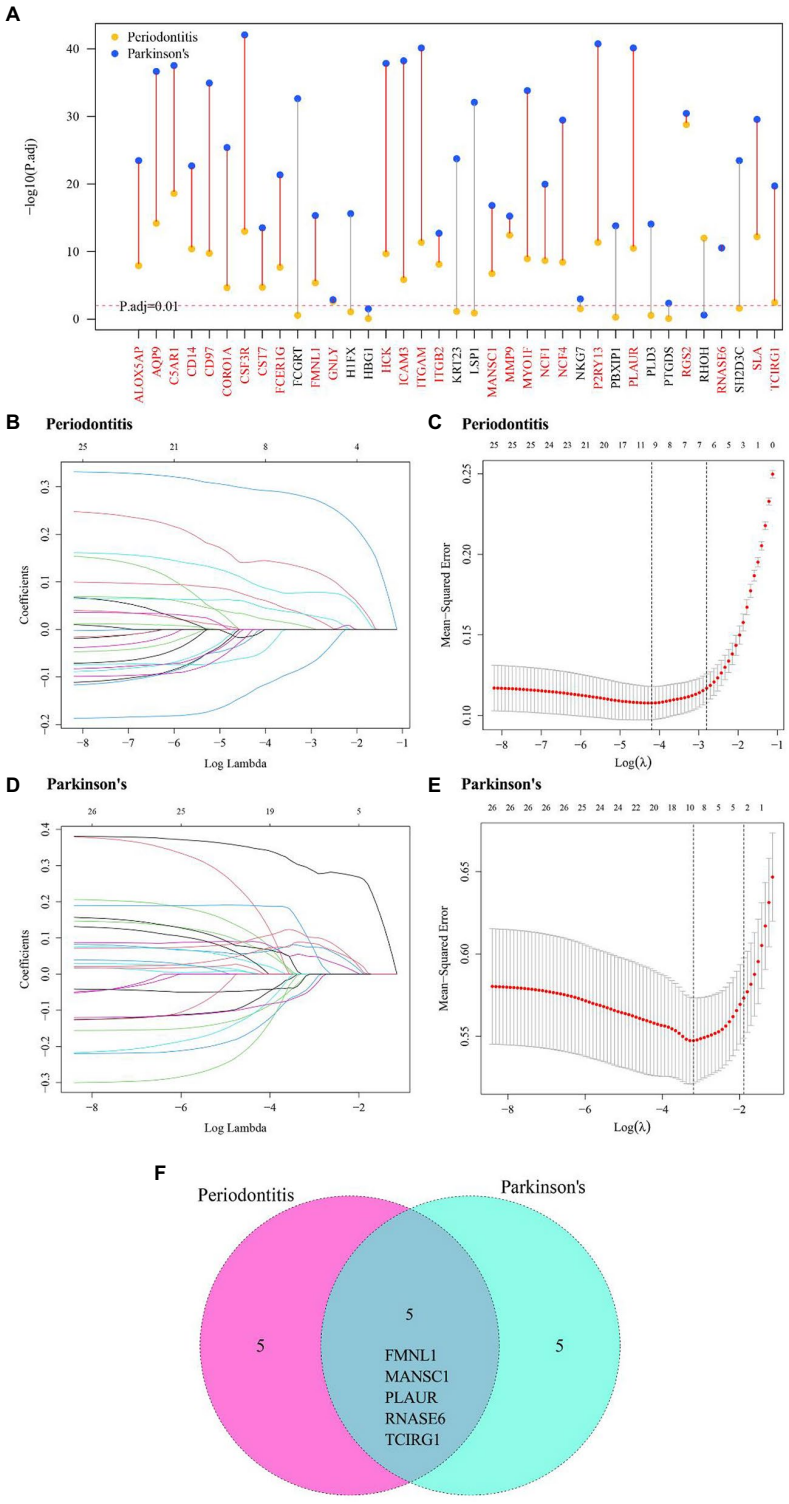
Hub cross-talk gene screening. **(A)** DE-cluster cross-talk gene of periodontitis and Parkinson’s disease’s. **(B–D)** Results of LASSO analysis for periodontitis and Parkinson’s disease. Each line in the graph represents a gene, and a larger value of the log lambda as the gene tends to 0 indicates that the gene is more critical. **(C–E)** cross-validation results of Periodontitis disease and Parkinson’s disease model. **(F)** Periodontitis disease and Parkinson’s disease’s hub cross-talk genes.

A multi-group differential expression analysis of cross-talk genes were carried out based on the clusters of two diseases, and genes with P.adjust < 0.01 were selected to be the DE-cluster cross-talk genes. The DE-cluster cross-talk genes for both diseases were overlapped and thereby 26 common DE-cluster cross-talk genes were obtained (Figure 8A). Based on the clusters of both diseases, LASSO regression analysis was used to further screen the DE-cluster cross-talk genes (Figures 8B–E). After further screening for DE-cluster cross-talk genes common to both diseases, 5 Hub cross-talk genes (i.e., FMNL1, MANSC1, PLAUR, RNASE6, TCIRG1) were obtained (Figure 8F).

### Correlation between hub cross-talk genes and hub IRRGs

**Figure 9:**
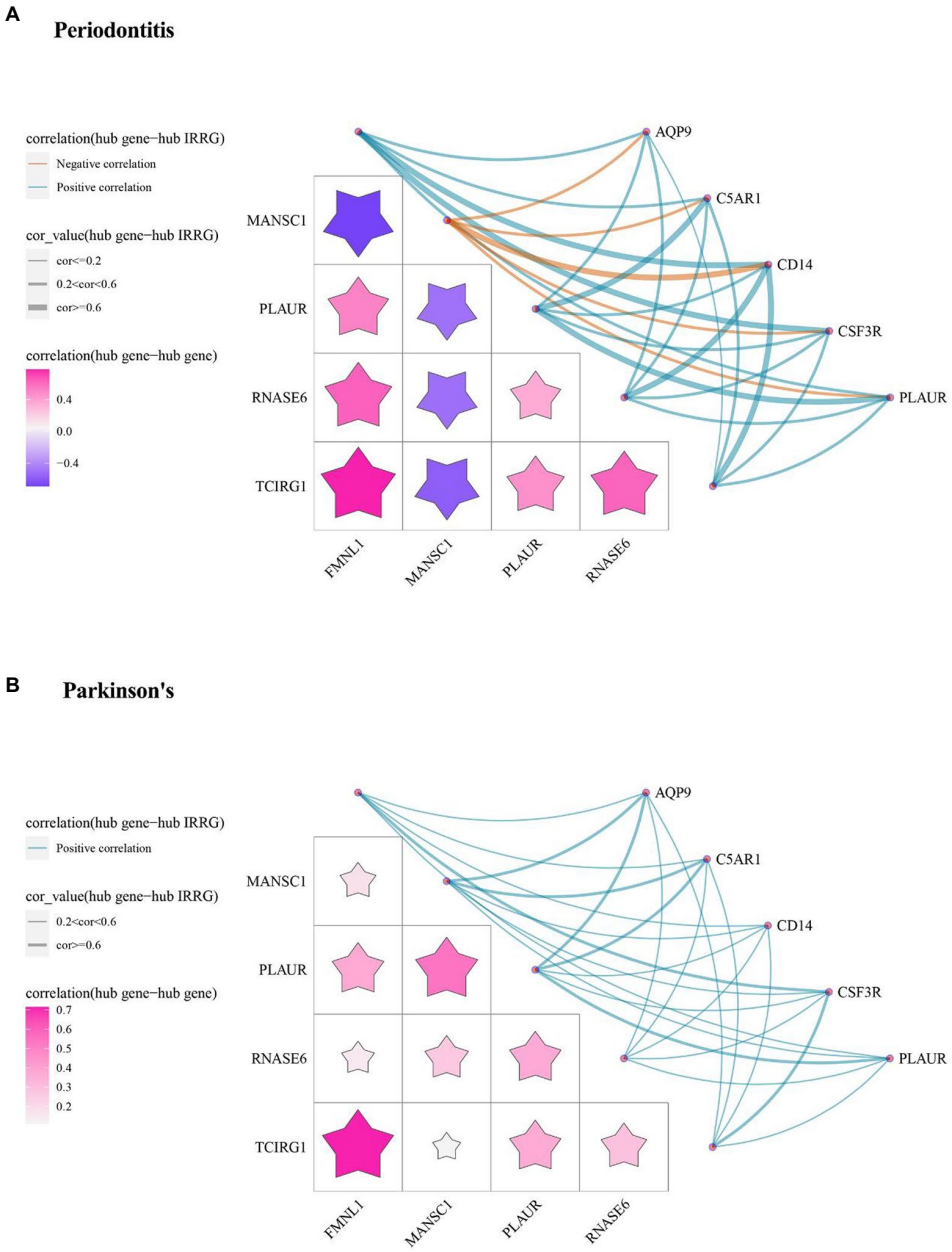
The relationships among Hub cross-talk genes and Hub IRRG in **(A)** Periodontitis disease and **(B)** Parkinson’s disease. The matrix graph in the lower left part of the figure shows the correlation coefficient between the hub cross-talk gene and the hub cross-talk gene, and the upper right part of the network diagram shows the correlation coefficient between the hub cross-talk gene and the hub IRRG.

The inflammatory response-related cross-talk genes were obtained and 5 genes (i.e., AQP9, C5AR1, CD14, CSF3R, PLAUR) were labeled as hub IRRGs. The expression values of 5 hub IRRGs and 5 hub cross-talk genes in the case sample of periodontitis and PD were obtained. Afterward, the relationship among the hub cross-talk genes, as well as the relationship between the hub cross-talk genes and the hub IRRGs were analyzed by using the Pearson correlation coefficient analysis (Figures 9A,B; Tables 5–6). MANSC1 was significantly negatively correlated with 4 hub cross-talk genes in periodontitis, and MANSC1 was significantly negatively correlated with 5 hub IRRGs. There was a significant positive correlation among the remaining 4 hub cross-talk genes, and the remaining 4 Hub cross-talk genes were also significantly positively correlated with 5 Hub IRRGs (Figure 9A). Five hub cross-talk genes were significantly positively correlated with five hub IRRGs in PD. Among these correlation, the correlation between CD14 and five hub cross-talk genes showed comparatively higher correlation (Figure 9B).

### Hub cross-talk genes’ expression level analysis

**Figure 10:**
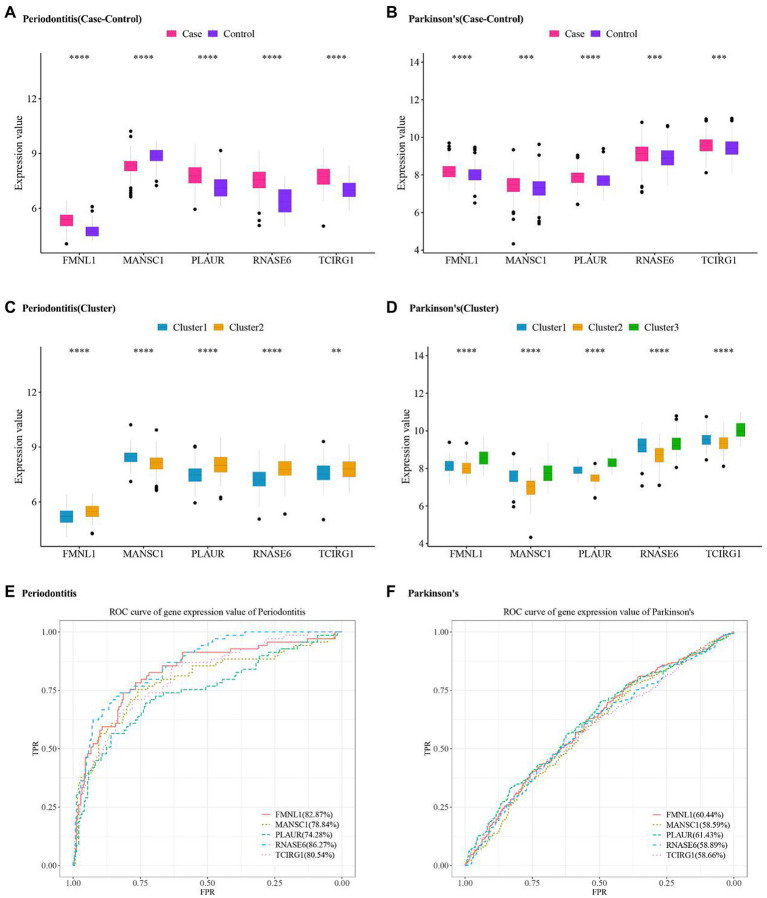
Hub cross-talk gene expression level and ROC analysis in periodontitis and Parkinson’s disease. **(A,B)** Hub cross-talk gene expression levels and differences between disease samples and normal samples in periodontitis and Parkinson’s disease, respectively. **(C,D)** Hub cross-talk gene expresses levels and differences in different clusters of periodontitis and Parkinson’s disease. The smaller the test result, the more “*” on the plot, and the correspondence between the *p* value and the “*” sign is ns: *p* > 0.05, *: *p* ≤ 0.05, **: *p* ≤ 0.01, ***: *p* ≤ 0.001, ****: *p* ≤ 0.0001. **(E,F)** ROC analysis results regarding the diagnostic accuracy of hub cross-talk genes in periodontitis and Parkinson’s disease.

The expression values of five hub cross-talk genes in periodontitis and PD were obtained. The Wilcoxon test results showed that five hub cross-talk genes had significant differences between disease samples and normal samples (Figures 10A,B). Afterward, the expression matrix of the five hub cross-talk genes in different clusters were obtained. The Wilcoxon test and Kruskal test results showed that the five hub cross-talk genes had significant differences between different cluster samples (Figures 10C,D). Furthermore, ROC analysis results showed that the AUC values of all five hub cross-talk gene in periodontitis were greater than 70% (Figure 10E). The AUC values of the five hub cross-talk gene in PD were greater than 58%. The AUC values of FMNL1 and PLAUR in PD were comparatively higher compared to the remaining three hub cross-talk genes (Figure 10F).

### Immune infiltration analysis of periodontitis and PD

**Figure 11:**
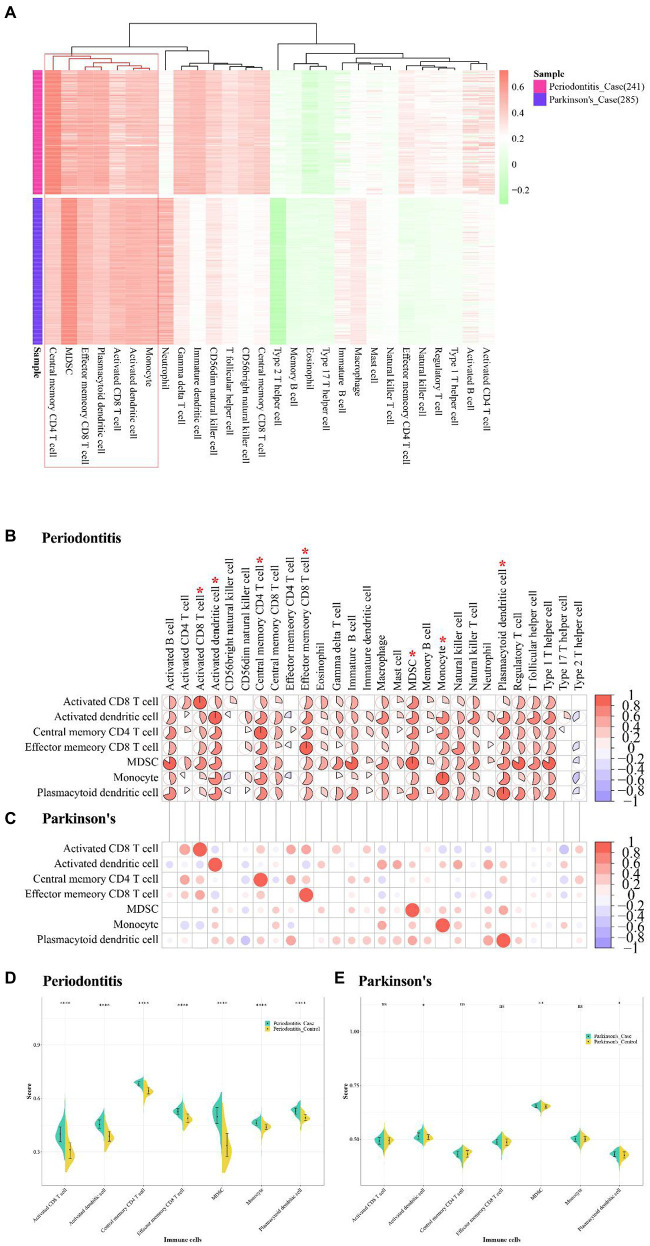
Immune infiltrative analysis of Periodontitis disease and Parkinson’s disease. **(A)** The abundance fraction of immune cells in disease samples of Periodontitis disease and Parkinson’s disease. **(B,C)** Correlation of higher abundance immune cells and other immune cells in Periodontitis disease and Parkinson’s disease. **(D,E)** Immune cell enrichment fractions in different sample types of Periodontitis and Parkinson’s disease.

The immune cell-related dataset were obtained from TISDB database, which included 782 genes and 28 types of immune cells. The immune infiltration quantification analysis was performed by using the ssGSEA algorithm, and the enrichment fractions of immune cells in periodontitis and PD were obtained. The “pheamap” package of R was used to demonstrate immune-infiltrating cell scores for case samples in periodontitis and PD. The results showed that central memory CD4 T cell, MDSC, Effector memory CD8 T cell, plasmacytoid dendritic cell, activated CD8 T cell, activated dendritic cell and monocyte were highly expressed in periodontitis and PD (Figures 11A,B).

The correlation between these high-abundance immune cells and other immune cells were analyzed by using the Pearson correlation coefficient, and the immune cells with value of *p* <0.05 were visualized (Table 7). The results showed that MDSC was highly positively related with active B cell, type 1 T helper cell, regulatory T cell, and immature B cell in periodontitis (cor > 0.8; Figure 11C). Activated CD4 T cell and Activated CD8 T cell were positively correlated in PD (cor = 0.6; Figure 11D). The active CD4 T cell was positively correlated with activated CD8 T cell in periodontitis.

**Table 7:** The correlation between immune cells in periodontitis and Parkinson’s disease.

Immune cell Type 1	Immune cell Type 2	*r-*value	*p*-value	Significance
**Periodontitis**				
Activated B cell	MDSC	0.84697554	1.60E-67	***
Type 1 T helper cell	MDSC	0.840122561	1.95E-65	***
Regulatory T cell	MDSC	0.837361374	1.26E-64	***
Immature B cell	MDSC	0.816824025	5.01E-59	***
Activated dendritic cell	Monocyte	0.764481625	1.80E-47	***
Monocyte	Activated dendritic cell	0.764481625	1.80E-47	***
Activated dendritic cell	Plasmacytoid dendritic cell	0.722465839	3.53E-40	***
Plasmacytoid dendritic cell	Activated dendritic cell	0.722465839	3.53E-40	***
Central memory CD4 T cell	Monocyte	0.714514793	6.01E-39	***
Monocyte	Central memory CD4 T cell	0.714514793	6.01E-39	***
T follicular helper cell	Activated dendritic cell	0.711295204	1.84E-38	***
MDSC	Plasmacytoid dendritic cell	0.707824783	6.05E-38	***
Plasmacytoid dendritic cell	MDSC	0.707824783	6.05E-38	***
Natural killer cell	Effector memory CD8 T cell	0.706920024	8.23E-38	***
Activated B cell	Plasmacytoid dendritic cell	0.705392312	1.38E-37	***
Activated dendritic cell	MDSC	0.697836471	1.69E-36	***
MDSC	Activated dendritic cell	0.697836471	1.69E-36	***
Macrophage	MDSC	0.686624343	6.05E-35	***
Macrophage	Activated dendritic cell	0.67722499	1.08E-33	***
Central memory CD4 T cell	MDSC	0.66604896	2.87E-32	***
MDSC	Central memory CD4 T cell	0.66604896	2.87E-32	***
T follicular helper cell	MDSC	0.664832249	4.07E-32	***
Activated dendritic cell	Central memory CD4 T cell	0.657279808	3.43E-31	***
Central memory CD4 T cell	Activated dendritic cell	0.657279808	3.43E-31	***
Natural killer T cell	Activated dendritic cell	0.653593529	9.49E-31	***
Type 1 T helper cell	Activated dendritic cell	0.65032303	2.31E-30	***
Activated CD8 T cell	MDSC	0.644174127	1.20E-29	***
MDSC	Activated CD8 T cell	0.644174127	1.20E-29	***
Natural killer T cell	MDSC	0.64259343	1.82E-29	***
Immature B cell	Plasmacytoid dendritic cell	0.637325431	7.19E-29	***
Central memory CD4 T cell	Effector memory CD8 T cell	0.636332538	9.29E-29	***
Effector memory CD8 T cell	Central memory CD4 T cell	0.636332538	9.29E-29	***
Monocyte	Plasmacytoid dendritic cell	0.618419171	8.02E-27	***
Plasmacytoid dendritic cell	Monocyte	0.618419171	8.02E-27	***
Central memory CD4 T cell	Plasmacytoid dendritic cell	0.61554209	1.60E-26	***
Plasmacytoid dendritic cell	Central memory CD4 T cell	0.61554209	1.60E-26	***
Activated dendritic cell	Effector memory CD8 T cell	0.612911102	2.98E-26	***
Effector memory CD8 T cell	Activated dendritic cell	0.612911102	2.98E-26	***
Activated B cell	Central memory CD4 T cell	0.609622487	6.46E-26	***
Gamma delta T cell	MDSC	0.609155618	7.20E-26	***
Type 1 T helper cell	Plasmacytoid dendritic cell	0.606999798	1.19E-25	***
Regulatory T cell	Plasmacytoid dendritic cell	0.602434095	3.39E-25	***
Effector memory CD8 T cell	Monocyte	0.595426389	1.64E-24	***
Monocyte	Effector memory CD8 T cell	0.595426389	1.64E-24	***
Activated B cell	Activated dendritic cell	0.582637192	2.64E-23	***
Type 1 T helper cell	Activated CD8 T cell	0.582132066	2.93E-23	***
Type 1 T helper cell	Central memory CD4 T cell	0.58162473	3.27E-23	***
Regulatory T cell	Activated dendritic cell	0.58144374	3.39E-23	***
Type 1 T helper cell	Effector memory CD8 T cell	0.574544534	1.44E-22	***
Eosinophil	MDSC	0.572134369	2.36E-22	***
Activated CD4 T cell	Activated CD8 T cell	0.569621164	3.94E-22	***
Effector memory CD8 T cell	MDSC	0.569618568	3.94E-22	***
MDSC	Effector memory CD8 T cell	0.569618568	3.94E-22	***
Effector memory CD8 T cell	Plasmacytoid dendritic cell	0.563135876	1.45E-21	***
Plasmacytoid dendritic cell	Effector memory CD8 T cell	0.563135876	1.45E-21	***
Natural killer cell	MDSC	0.561420539	2.04E-21	***
Natural killer cell	Plasmacytoid dendritic cell	0.552501589	1.16E-20	***
Regulatory T cell	Activated CD8 T cell	0.549123737	2.21E-20	***
T follicular helper cell	Activated CD8 T cell	0.54876323	2.36E-20	***
Activated CD8 T cell	Effector memory CD8 T cell	0.545321557	4.52E-20	***
Effector memory CD8 T cell	Activated CD8 T cell	0.545321557	4.52E-20	***
Central memory CD8 T cell	MDSC	0.54186573	8.60E-20	***
Natural killer T cell	Plasmacytoid dendritic cell	0.541209893	9.71E-20	***
MDSC	Monocyte	0.54120915	9.71E-20	***
Monocyte	MDSC	0.54120915	9.71E-20	***
Central memory CD8 T cell	Activated dendritic cell	0.541083811	9.93E-20	***
Central memory CD8 T cell	Central memory CD4 T cell	0.540839323	1.04E-19	***
Gamma delta T cell	Plasmacytoid dendritic cell	0.531789461	5.38E-19	***
T follicular helper cell	Monocyte	0.531104362	6.09E-19	***
Immature B cell	Activated CD8 T cell	0.529705171	7.81E-19	***
Regulatory T cell	Central memory CD4 T cell	0.528671052	9.38E-19	***
Activated B cell	Effector memory CD8 T cell	0.528579509	9.54E-19	***
Type 1 T helper cell	Monocyte	0.504458027	5.80E-17	***
Macrophage	Plasmacytoid dendritic cell	0.500397599	1.12E-16	***
Activated B cell	Activated CD8 T cell	0.499708365	1.25E-16	***
T follicular helper cell	Effector memory CD8 T cell	0.497187741	1.88E-16	***
Eosinophil	Activated CD8 T cell	0.485978771	1.09E-15	***
Immature B cell	Central memory CD4 T cell	0.485824724	1.12E-15	***
T follicular helper cell	Plasmacytoid dendritic cell	0.485378278	1.20E-15	***
Natural killer cell	Activated dendritic cell	0.48420841	1.43E-15	***
Natural killer T cell	Activated CD8 T cell	0.475167393	5.60E-15	***
Natural killer cell	Central memory CD4 T cell	0.472655726	8.13E-15	***
Central memory CD8 T cell	Monocyte	0.471236629	1.00E-14	***
Activated CD4 T cell	MDSC	0.463909411	2.90E-14	***
Immature B cell	Effector memory CD8 T cell	0.46250849	3.54E-14	***
Activated B cell	Monocyte	0.460718763	4.57E-14	***
Macrophage	Activated CD8 T cell	0.457572029	7.13E-14	***
Natural killer T cell	Effector memory CD8 T cell	0.456250217	8.58E-14	***
Natural killer cell	Monocyte	0.451913064	1.57E-13	***
Macrophage	Monocyte	0.447029978	3.05E-13	***
Regulatory T cell	Effector memory CD8 T cell	0.444491073	4.30E-13	***
T follicular helper cell	Central memory CD4 T cell	0.441533585	6.40E-13	***
Central memory CD8 T cell	Plasmacytoid dendritic cell	0.439049415	8.89E-13	***
Activated CD8 T cell	Activated dendritic cell	0.436269671	1.28E-12	***
Activated dendritic cell	Activated CD8 T cell	0.436269671	1.28E-12	***
CD56dim natural killer cell	Activated dendritic cell	0.433192287	1.91E-12	***
Activated CD8 T cell	Central memory CD4 T cell	0.43163635	2.34E-12	***
Central memory CD4 T cell	Activated CD8 T cell	0.43163635	2.34E-12	***
Immature B cell	Activated dendritic cell	0.426458372	4.54E-12	***
Gamma delta T cell	Activated dendritic cell	0.420832272	9.21E-12	***
Mast cell	MDSC	0.414996797	1.89E-11	***
Gamma delta T cell	Central memory CD4 T cell	0.407666833	4.58E-11	***
Macrophage	Central memory CD4 T cell	0.400362875	1.08E-10	***
Neutrophil	Activated dendritic cell	0.367303528	4.12E-09	***
Gamma delta T cell	Activated CD8 T cell	0.366367009	4.54E-09	***
Natural killer T cell	Central memory CD4 T cell	0.363634497	6.02E-09	***
CD56dim natural killer cell	Monocyte	0.363021235	6.41E-09	***
Macrophage	Effector memory CD8 T cell	0.353705384	1.64E-08	***
Immature dendritic cell	Central memory CD4 T cell	0.353607436	1.66E-08	***
Immature dendritic cell	Activated dendritic cell	0.349212099	2.56E-08	***
Natural killer T cell	Monocyte	0.345840064	3.55E-08	***
Eosinophil	Plasmacytoid dendritic cell	0.34454255	4.02E-08	***
Natural killer cell	Activated CD8 T cell	0.344293922	4.12E-08	***
CD56dim natural killer cell	Effector memory CD8 T cell	0.338007281	7.48E-08	***
Neutrophil	Plasmacytoid dendritic cell	0.336720129	8.44E-08	***
Regulatory T cell	Monocyte	0.327379534	1.99E-07	***
Activated CD8 T cell	Plasmacytoid dendritic cell	0.323496414	2.83E-07	***
Plasmacytoid dendritic cell	Activated CD8 T cell	0.323496414	2.83E-07	***
Eosinophil	Effector memory CD8 T cell	0.320532888	3.68E-07	***
Eosinophil	Central memory CD4 T cell	0.302555673	1.71E-06	***
**Parkinson’s disease**				
Activated CD4 T cell	Activated CD8 T cell	0.599074977	3.77E-29	***
Neutrophil	Plasmacytoid dendritic cell	0.500181503	1.90E-19	***
Effector memory CD4 T cell	Plasmacytoid dendritic cell	0.484410676	3.56E-18	***
Activated CD4 T cell	Central memory CD4 T cell	0.476766534	1.40E-17	***
Macrophage	Activated dendritic cell	0.473049766	2.68E-17	***
Activated CD8 T cell	Effector memory CD8 T cell	0.456873144	4.20E-16	***
Effector memory CD8 T cell	Activated CD8 T cell	0.456873144	4.20E-16	***
Effector memory CD4 T cell	Activated CD8 T cell	0.443595434	3.61E-15	***
MDSC	Plasmacytoid dendritic cell	0.435338298	1.31E-14	***
Plasmacytoid dendritic cell	MDSC	0.435338298	1.31E-14	***
Neutrophil	Activated dendritic cell	0.433717488	1.68E-14	***
Natural killer cell	Activated dendritic cell	0.4192127	1.48E-13	***
Effector memory CD4 T cell	Central memory CD4 T cell	0.416668771	2.14E-13	***
Macrophage	Monocyte	0.405530203	1.04E-12	***
Mast cell	Activated dendritic cell	0.400738176	2.03E-12	***
Mast cell	Plasmacytoid dendritic cell	0.388706161	1.03E-11	***
Gamma delta T cell	Activated CD8 T cell	0.384135928	1.87E-11	***
Type 2 T helper cell	Central memory CD4 T cell	0.363258068	2.57E-10	***
Immature dendritic cell	Plasmacytoid dendritic cell	0.351256754	1.07E-09	***
Activated CD8 T cell	Central memory CD4 T cell	0.349402777	1.32E-09	***
Central memory CD4 T cell	Activated CD8 T cell	0.349402777	1.32E-09	***
Central memory CD4 T cell	Effector memory CD8 T cell	0.340048326	3.82E-09	***
Effector memory CD8 T cell	Central memory CD4 T cell	0.340048326	3.82E-09	***
Macrophage	Plasmacytoid dendritic cell	0.333212561	8.11E-09	***
Gamma delta T cell	Plasmacytoid dendritic cell	0.330016388	1.15E-08	***
Natural killer cell	Monocyte	0.326552107	1.66E-08	***
Immature dendritic cell	Activated CD8 T cell	0.310533819	8.69E-08	***
Type 2 T helper cell	Activated CD8 T cell	0.309078509	1.00E-07	***

***p <= 0.001.

The violin plots were used to show the fractional distribution of high-abundance immune cells in periodontitis and PD, and the Wilcoxon test was applied to observe the significance of samples. The results showed that the high abundance of immune cells varied greatly between case samples and normal samples in periodontitis (Figure 11D). The high-abundance immune cells differed less between case samples and normal samples in PD (Figure 11E). The effector memory CD8 T cell was highly enriched in both periodontitis and PD.

### Relationship between high-abundance immune cells and hub cross-talk genes

**Figure 12:**
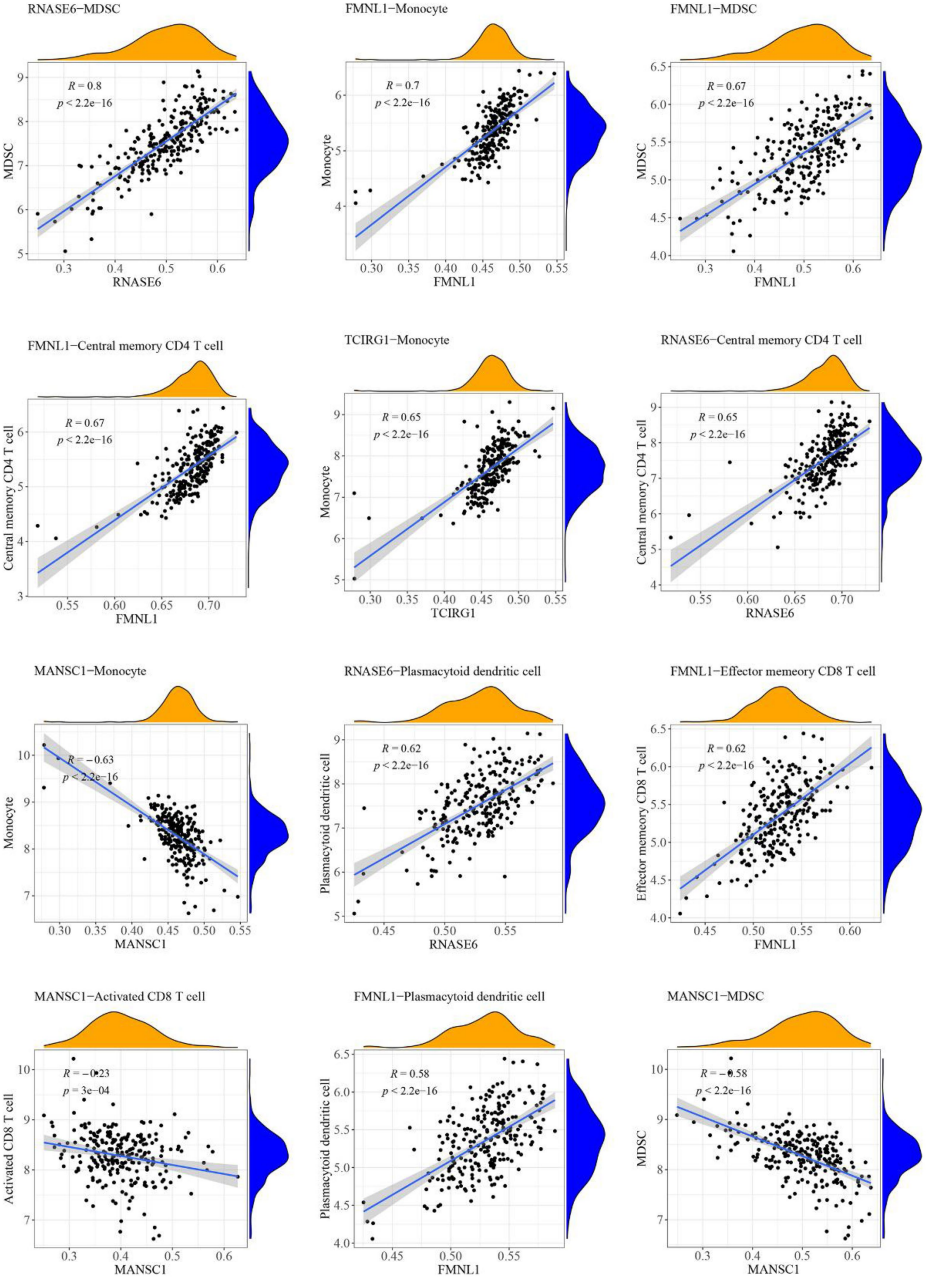
Relationship between high-abundance immune cells and hub cross-talk genes in periodontitis.

**Figure 13:**
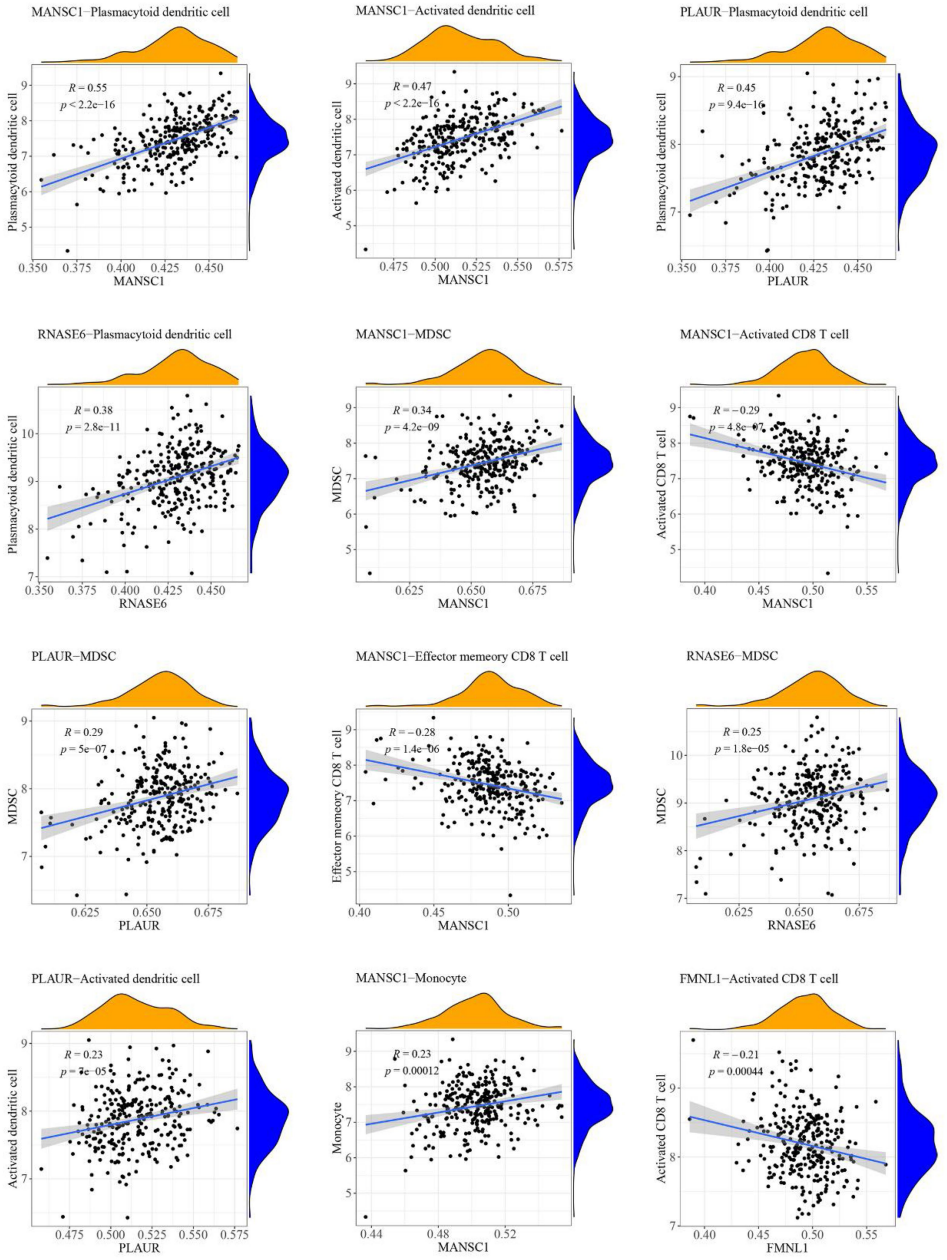
Relationship between high-abundance immune cells and hub cross-talk gene in Parkinson’s disease.

The case sample scores for 7 high-abundance immune cells and 5 hub cross-talk genes were obtained. The correlation analysis was carried out to invesitgate the correlation between immune cells and hub cross-talk genes. The relationship pairs with value of *p* < 0.05 was defined to be significant. The absolute value of correlation coefficient was calculated and the results of the top12 were visualized. The results shwoed that RNASE6 gene was highly correlated with MDSC in periodontitis (Figure 12). MANSC1 was highly correlated with plasmacytoid dendritic cell in PD (Figure 13; Table 8).

**Table 8:** The correlation between hub crosstalk genes and immune cells in periodontitis and Parkinson’s disease, respectively.

Hub crosstalk genes	Immune cells	*r*-value	abs_r value	*p*-value	Significance
**Periodontitis**					
RNASE6	MDSC	0.804313666	8.04E-01	5.98E-56	***
FMNL1	Monocyte	0.701080924	7.01E-01	5.82E-37	***
FMNL1	MDSC	0.674505139	6.75E-01	2.42E-33	***
FMNL1	Central memory CD4 T cell	0.668905401	6.69E-01	1.26E-32	***
TCIRG1	Monocyte	0.651103432	6.51E-01	1.87E-30	***
RNASE6	Central memory CD4 T cell	0.647773522	6.48E-01	4.60E-30	***
RNASE6	Plasmacytoid dendritic cell	0.620123162	6.20E-01	5.31E-27	***
FMNL1	Effector memory CD8 T cell	0.618680488	6.19E-01	7.53E-27	***
FMNL1	Plasmacytoid dendritic cell	0.582572461	5.83E-01	2.67E-23	***
TCIRG1	Central memory CD4 T cell	0.555225112	5.55E-01	6.85E-21	***
FMNL1	Activated dendritic cell	0.55205012	5.52E-01	1.26E-20	***
PLAUR	Activated dendritic cell	0.532123801	5.32E-01	5.07E-19	***
TCIRG1	MDSC	0.506582068	5.07E-01	4.09E-17	***
RNASE6	Activated dendritic cell	0.468481658	4.68E-01	1.50E-14	***
RNASE6	Activated CD8 T cell	0.446647646	4.47E-01	3.22E-13	***
PLAUR	Monocyte	0.443562776	4.44E-01	4.88E-13	***
RNASE6	Monocyte	0.437623294	4.38E-01	1.07E-12	***
TCIRG1	Activated dendritic cell	0.427129687	4.27E-01	4.17E-12	***
TCIRG1	Plasmacytoid dendritic cell	0.420248526	4.20E-01	9.90E-12	***
TCIRG1	Effector memory CD8 T cell	0.40759104	4.08E-01	4.62E-11	***
PLAUR	Effector memory CD8 T cell	0.383329366	3.83E-01	7.43E-10	***
RNASE6	Effector memory CD8 T cell	0.376706992	3.77E-01	1.53E-09	***
FMNL1	Activated CD8 T cell	0.346157886	3.46E-01	3.44E-08	***
PLAUR	Plasmacytoid dendritic cell	0.345852593	3.46E-01	3.54E-08	***
PLAUR	Central memory CD4 T cell	0.342304629	3.42E-01	4.98E-08	***
PLAUR	MDSC	0.318805279	3.19E-01	4.28E-07	***
TCIRG1	Activated CD8 T cell	0.234786142	0.234786142	0.000235579	***
MANSC1	Activated CD8 T cell	−0.231175404	0.231175404	0.000295404	***
MANSC1	Central memory CD4 T cell	−0.529187133	5.29E-01	8.56E-19	***
MANSC1	Effector memory CD8 T cell	−0.532649636	5.33E-01	4.61E-19	***
MANSC1	Plasmacytoid dendritic cell	−0.539617258	5.40E-01	1.30E-19	***
MANSC1	MDSC	−0.576288835	5.76E-01	1.00E-22	***
MANSC1	Activated dendritic cell	−0.60804292	6.08E-01	9.33E-26	***
MANSC1	Monocyte	−0.625435826	6.25E-01	1.45E-27	***
**Parkinson’s disease**					
MANSC1	Plasmacytoid dendritic cell	0.551790642	0.551790642	4.15E-24	***
MANSC1	Activated dendritic cell	0.467804822	0.467804822	6.65E-17	***
PLAUR	Plasmacytoid dendritic cell	0.451944976	0.451944976	9.43E-16	***
RNASE6	Plasmacytoid dendritic cell	0.381089523	0.381089523	2.77E-11	***
MANSC1	MDSC	0.33920803	0.33920803	4.19E-09	***
PLAUR	MDSC	0.292493364	0.292493364	4.99E-07	***
RNASE6	MDSC	0.251153249	0.251153249	1.78E-05	***
PLAUR	Activated dendritic cell	0.233308169	0.233308169	7.00E-05	***
MANSC1	Monocyte	0.226357563	0.226357563	0.000115866	***
PLAUR	Monocyte	0.183419906	0.183419906	0.001875335	**
TCIRG1	Effector memory CD8 T cell	0.169561529	0.169561529	0.004094811	**
RNASE6	Activated CD8 T cell	0.167950803	0.167950803	0.004467738	**
FMNL1	Monocyte	0.143364384	0.143364384	0.015428656	*
TCIRG1	Monocyte	0.143009576	0.143009576	0.015687724	*
RNASE6	Monocyte	0.131393959	0.131393959	0.026550632	*
TCIRG1	Activated dendritic cell	−0.120360896	0.120360896	0.042317655	*
FMNL1	Central memory CD4 T cell	−0.12064161	0.12064161	0.041835486	*
PLAUR	Activated CD8 T cell	−0.123935985	0.123935985	0.036514285	*
FMNL1	MDSC	−0.143148162	0.143148162	0.015586084	*
FMNL1	Activated CD8 T cell	−0.206836013	0.206836013	0.000440536	***
MANSC1	Effector memory CD8 T cell	−0.281186359	0.281186359	1.41E-06	***
MANSC1	Activated CD8 T cell	−0.292814914	0.292814914	4.85E-07	***

*p <= 0.05,**p <= 0.01, ***p <= 0.001.

### Hub cross-talk gene PPI-pathway network

**Figure 14:**
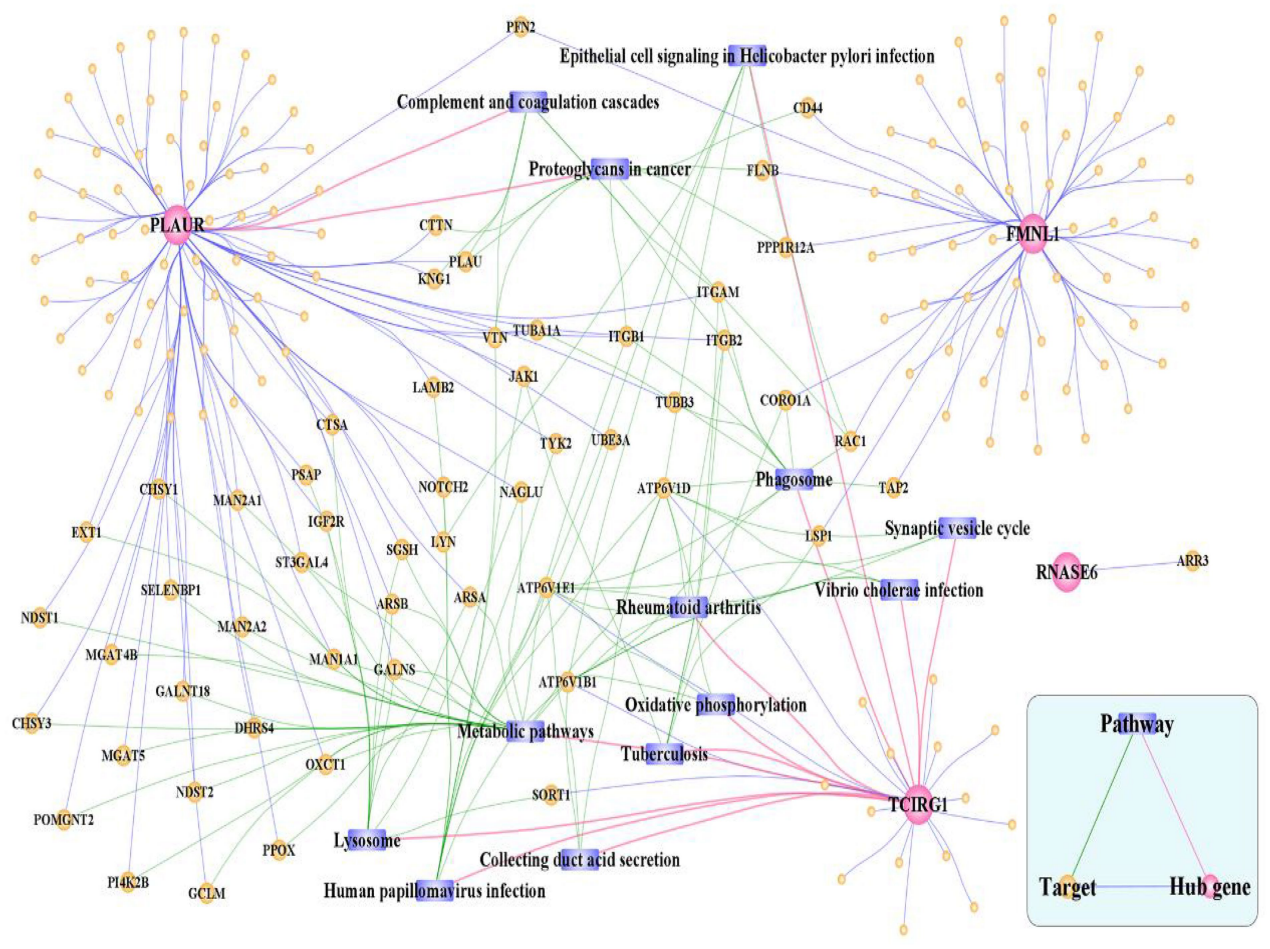
Hub cross-talk gene related complex network. The network consists of 210 nodes and 303 edges.

The 192 hub cross-talk gene-target interaction pairs were obtained from the HPRD and BIOGRID database. Based on the KEGG database, 105 hub cross-talk gene-pathway-target interaction pairs were obtained. The Cytoscape software was used to integrate the hub cross-talk gene-target pairs with the hub cross-talk gene-pathway-target interaction pairs; thereby a hub cross-talk gene related complex network was constructed (Figure 14). Figure 14 shows that PLAUR interacted with other genes and regulated the composite and coagulation cascades pathway. TCIRG1 interacted with other hub cross-talk genes and was involved in regulating oxidative phosphorylation pathway and phagosome pathway.

## Discussion

The main findings of the current study identified five hub crosstalk genes (i.e., FMNL1, MANSC1, PLAUR, RNASE6, and TCIRG1) to be the linking mechanisms between periodontitis and PD. In addition, this study also identified several signaling pathways enriched by the crosstalk genes, for example, complement and coagulation cascades, neutrophil extracellular trap formation, transendothelial leukocyte migration, and phagosome formation. In this section, previous literature were reviewed to explain the linking role of these genetic factors in the pathogenesis of both diseases.

The formin FRL1 (FMNL1) resides in the actin-rich cores of primary macrophage podosomes. During adhesion and migration of macrophages, FMNL1 is responsible for modifiying actin at the podosome in macrophages (Mersich et al., 2010). By stabilizing podosome lifespans without interacting with fast-growing actin termini, FMNL1 promotes migration and recruitment activity of macrophages (Miller et al., 2017). Additionally, FMNL1 is upregulated in the course of monocyte differentiation to macrophages (Mersich et al., 2010), which might be used for explaining the significant correlation between FMNL1 and monocytes in periodontitis (Table 8: *r* = 0.701080924; *p* = 5.82E-37) and PD (Table 8: *r* = 0.143364384; *p* = 0.015428656). The potential role of FMNL1 in enhancing macrophage activity might be involved in the mechanism of periodontitis inducing PD progression. In periodontitis, overgrowth of Gram-negative bacteria and LPS access to the blood circulation may enhance the activation of macrophages in peripheral blood (Pussinen et al., 2004). One of the hallmarks of PD is the impairment of BBB integrity and function (Al-Bachari et al., 2020), due to which immune cells, including macrophage clusters, can infiltrate from the peripheral blood into the CNS (Su and Zhou, 2021). The periodontal pathogen, *Escherichia coli (E. coli)*, can also penetrate the impaired BBB of a PD patient. In a study, the LPS extracted from the cell wall of *E. coli* was found to induce rapid and intense activation of microglia and macrophages (Blaylock, 2017). The macrophages stimulated by LPS caused robust neurotoxicity and immunoexcitotoxicity, which may play a central role in PD-associated neurodegeneration progression (Blaylock, 2017). Based on these studies, it may be suggested that the crosstalk gene FMNL1 is a common link between periodontitis and PD *via* macrophage activation. However, there is no published research investigating the deregulation and function of FMNL1 in linking periodontitis and PD to date.

The Plasminogen Activator Urokinase (PLAU) gene encodes the receptor for Urokinase-type Plasminogen Activator (uPA). uPA has a role in localizing and promoting plasmin formation and was found to be implicated in the pathological processes associated with cell-surface plasminogen activation and localized degradation of the extracellular matrix. In periodontitis, uPA converts plasminogen to plasmin, leading to the uPA proteolytic cascade activated by *P. gingivalis* and inducing tissue destruction, particularly, alveolar bone loss (Fleetwood et al., 2015). Additionally, uPA was found to be a marker of macrophage activation based on its role in regulating macrophage motility and macrophage-mediated matrix degradation in periodontitis (Fleetwood et al., 2014). In PD, uPA was found to act as an activator protease in the process of plasminogen activation and upregulate the expression of the serine protease inhibitor (serpin) plasminogen activator inhibitor-1 (PAI-1), leading to neuroinflammation (Reuland and Church, 2020). Further, an increased expression of Urokinase Plasminogen-activator Receptor (uPAR) was observed in activated microglia in the brain of a patient with another neurodegenerative disease (Alzheimer’s disease), and uPAR expression was found to be mediated by oxidative stress-related mechanisms (Walker et al., 2002).

TCIRG1 (T Cell Immune Regulator 1, ATPase H+ Transporting V0) encodes the a3 isoform of V-ATPase a subunit, which is essential for osteoclastic bone resorption. The promoter activity of serial-deletion fragments of the TCIRG1 gene promoter was observed to be enhanced throughout the osteoclastic differentiation process of osteoclast RAW264.7 cells, and such alteration was found to be induced by the receptor activator of nuclear factor kB ligand (RANKL) (Beranger et al., 2007). TCIRG1 expression was found to induce the bone resorption activity of osteoclasts in periodontal disease (Heo et al., 2022). TCIRG1 was highly expressed in myeloid cells and involved in myeloid cell activation and microglial neuroinflammation (Funatsu et al., 2022). The mRNA expression of TCIRG1 gene was recently examined in human alloactivated T lymphocytes, which indicates that TCIRG1 was essential in T cell activation (Heinemann et al., 1999). Our present study found that the TCIRG1 expression level was positively correlated with effector memory cluster of differentiation (CD)8+ T cells (*r* = 0.41; *p* = 4.62E-11) in periodontitis. Similarly, a positive and significant correlation was found between the TCIRG1 expression level and effector memory CD8+ T cells (*r* = 0.17; *p* = 0.004) in PD. In periodontitis, the CD8+ T cells with an effector memory phenotype were shown to release anti-inflammatory cytokines (interleukin [IL]-10 and transforming growth factor [TGF]-β) and suppress bone-destructive cytokines (interferon [IFN]-γ and IL-17), and thus play a protective role for the alveolar bone (Cardoso and Arosa, 2017). In PD, the cytotoxic attack of a robust CD8+ T cell infiltration might initiate and propagate neuronal death and synucleinopathy by secreting cytolytic enzymes (granzymes A, B, and K) and/or pro-inflammatory cytokines (IFN-γ) (Galiano-Landeira et al., 2020). These studies validated the possibility of our findings that the hub crosstalk gene TCIRG1 links periodontitis and PD *via* regulating effector memory CD8+ T cells.

The complement and coagulation cascade pathway was found to be enriched by the crosstalk genes linking periodontitis and PD. The complement system plays a vital role in immune surveillance, homeostasis, and bridges the innate and adaptive immune systems (Ricklin et al., 2010). A study showed that suppressing component 3 (C3) in the complement cascade directly inhibits periodontal inflammation and indirectly counteracts dysbiosis, thus showing promising clinical potential for treating periodontitis (Hajishengallis et al., 2019). The complement C3-positive astrocytes were increased in the ventral midbrain of the intrastriatal α-synuclein preformed fibril (PFF)-injected mice, and C3 secreted from astrocytes could induce the degeneration of dopaminergic neurons, suggesting the potential involvement of complement and coagulation cascades in dopaminergic neurodegeneration in PD (Ma et al., 2021). Considering the involvement of complement system activation in both periodontitis and PD, this pathway might link both diseases by means of neuroimmune interaction. The complement system activation triggered by periodontitis may regulate the migration and invasion function of the peripheral immune cells (Merle et al., 2015), which may penetrate the damaged BBB and induce neuroinflammation and neurodegeneration in PD patients (Chao et al., 2014). Apart from the complement and coagulation cascade pathway, oxidative phosphorylation was identified to be an important pathogenic pathway contributing the linkages between both diseases. It is through oxygen oxidative phosphorylation that oxygen inhaled by the body is used to produce energy (Cole, 2016). Mutations in mitochondrial DNA result in reduced efficiency of oxidative phosphorylation and ATP production, overproduction of ROS, and a significant reduction in mitochondrial membrane potential (MMP) levels in pathophysiological conditions (Xu et al., 2021). The downregulation of PD-related gene-complex I was found to be involved in oxidative phosphorylation by leading to reduced ATP formation in neurons and further inducing neuronal apoptosis (Ali and Dholaniya, 2022). In addition, *P. gingivalis* infection was found to promote mitochondrial fragmentation and dysfunction, increase the levels of mitochondrial reactive oxygen species (mtROS), and upregulate the phosphorylation of Drp1 gene (Xu et al., 2021).

The current study found that several immune cells (e.g., central memory CD4 T cells, effector memory CD8 T cells, activated CD8 T cells, myeloid-derived suppressor cells (MDSCs), plasmacytoid dendritic cells, activated dendritic cells, and Monocytes) were highly expressed in periodontitis disease and PD. Among these immune cells, MDSCs and dendritic cells obtained our particular interest. It is believed that MDSCs have the most potential for restoring homeostasis after inflammation, as well as being able to suppress adaptive immunity by suppressing T cell response (Kauppinen et al., 2020). The MDSCs was detected to be significantly increased in peripheral blood of patients with PD compared with healthy control individuals, which led to the increased production of immunosuppression-related genes [arginase 1 (ARG1), interleukin-10 (IL-10), and cyclooxygenase 2 (COX-2)] (Yang et al., 2018). MDSCs were also found to be expanded, activated, and recruited as the result of the inflammatory response induced by *P. gingivalis* infection in periodontitis (Valero-Monroy et al., 2016; Su et al., 2017). As professional antigen-presenting cells, dendritic cells link innate and adaptive immunity and are vital to the induction of protective immune responses against pathogens (Liu et al., 2021). Peripheral blood dendritic cells in chronic periodontitis was found to carry *P. gingivalis*. Such microbial carriage state not only enhanced the differentiation of monocytes into immature myeloid dendritic cells, but also promoted the production of matrix metalloproteinase-9 and upregulated C1q, heat shock protein 60, heat shock protein 70, CCR2, and CXCL16 (Carrion et al., 2012). The recruitment of activated subsets of dendritic cells in the brain was found to increase the production of pro-inflammatory cytokines (e.g., TNFα, IL1β, and IL6) and aggregate alpha synuclein (Agg α-syn) fuels neuroinflammation in PD (Magnusen et al., 2018).

It is noteworthy to highlight the limitation of the current study. The genetic factors identified in the current study are obtained solely using computational prediction based on the periodontitis-and PD-associated datasets; however, these factors were not validated by performing related experiments. The representative periodontal pathogen *P. gingivalis* or its derived LPS could be used for infecting the microglia and its activated immune and inflammation-related signaling pathways could be examined. The transcriptomic alterations of microglial cells under such stimulation can be examined by performing next generation sequencing approach. The animal model with PD could be established to identify the influence of *P. gingivalis* or its derived LPS on the immune cells in the brain. Another limitation is regarding the GEO datasets analyzed in the current research. Although four datasets were included and analyzed in the current research, the sample size included in each dataset restricted the prediction accuracy of the results. In addition, there is no public dataset integrating both diseases and investigating the alteration in mRNA expression of the peripheral blood tissue in PD with/without periodontitis. Nevertheless, this study has potential for clinical application by suggesting putative genetic mechanisms underlying the increased risk of periodontitis in PD progression. The five hub crosstalk genes discussed in this study hold promise to be developed as a chair-side kit for predicting the risk of PD in elderly periodontitis patients. Further investigation is needed to validate the prediction accuracy of these genetic findings.

## Conclusion

Five genes (i.e., FMNL1, MANSC1, PLAUR, RNASE6, and TCIRG1) were identified as crosstalk biomarkers linking PD and periodontitis. The significant correlation between these crosstalk genes and immune cells strongly suggests the involvement of immunology in linking both diseases.

## Data availability statement

The original contributions presented in the study are included in the article/Supplementary material, further inquiries can be directed to the corresponding author.

## Author contributions

SHu and SL: conceptualization, funding acquisition, methodology, formal analysis, and writing—original draft. WN, XH, XL, YD, DF, AO, BL, VS, HL, SG, RZ, and DZ: methodology, formal analysis, review, and editing. GS, SHu, and SHuang: project administration and supervision. All authors contributed to the article and approved the submitted version.

## Funding

We appreciate the funding by the Science Research Cultivation Program of Stomatological Hospital, Southern Medical University (Grant No.: PY2022001 for supporting the postdoc research of Dr. Shaonan Hu (Email: shaonan_hu@smu.edu.cn) and Grant No.: PY2020004 for supporting the postdoc research of Dr. Simin Li (Email: simin.li.dentist@ gmail.com)).

## Conflict of interest

The authors declare that the research was conducted in the absence of any commercial or financial relationships that could be construed as a potential conflict of interest.

## Publisher’s note

All claims expressed in this article are solely those of the authors and do not necessarily represent those of their affiliated organizations, or those of the publisher, the editors and the reviewers. Any product that may be evaluated in this article, or claim that may be made by its manufacturer, is not guaranteed or endorsed by the publisher.

## Footnotes

^1^ http://www.ncbi.nlm.nih.gov/

^2^ https://www.gsea-msigdb.org/gsea/msigdb/human/geneset/HALLMARK_INFLAMMATORY_RESPONSE.html

^3^ https://www.gsea-msigdb.org/gsea/msigdb/

^4^ http://cis.hku.hk/TISIDB/download.php

^5^ http://www.hprd.org/

^6^ http://thebiogrid.org/

^7^ https://www.kegg.jp/
